# Characterization and Comparative Genomics Analysis of lncFII Multi-Resistance Plasmids Carrying *bla*_CTX__–__M_ and Type1 Integrons From *Escherichia coli*

**DOI:** 10.3389/fmicb.2021.753979

**Published:** 2021-11-16

**Authors:** Wei Zhou, Enbao Zhang, Jinzhi Zhou, Ze He, Yuqiao Zhou, Jianzhong Han, Daofeng Qu

**Affiliations:** ^1^Zhejiang Provincial Center for Animal Disease Prevention and Control, Hangzhou, China; ^2^Key Laboratory of Food Quality and Safety, School of Food Science and Biotechnology, Zhejiang Gongshang University, Hangzhou, China

**Keywords:** plasmid, β-Lactam antibiotics, *bla*_CTX__–__M_, MDR, mobile elements

## Abstract

This research aimed to investigate the presence and transferability of the extended-spectrum β-lactamase resistance genes to identify the genetic context of multi-drug resistant (MDR) loci in two *Escherichia coli* plasmids from livestock and poultry breeding environment. MICs were determined by broth microdilution. A total of 137 *E. coli* resistant to extended-spectrum β-lactam antibiotics were screened for the presence of the ESBL genes by PCR. Only two *E. coli* out of 206 strains produced carbapenemases, including strain 11011 that produced enzyme A, and strain 417957 that produced enzyme B. The genes were *bla*_KPC_ and *bla*_*NDM*_, respectively. The plasmids containing *bla*_CTX__–__M_ were conjugatable, and the plasmids containing carbapenem resistance gene were not conjugatable. Six extended-spectrum β-lactamase resistance genes were detected in this research, including *bla*_TEM_, *bla*_CTX__–__M_, *bla*_SHV_, *bla*_OAX__–__1_, *bla*_KPC_, and *bla*_*NDM*_, and the detection rates were 94.89% (130/137), 92.7% (127/137), 24.81% (34/137), 20.43% (28/137), 0.72% (1/137), and 0.72% (1/137), respectively. Two conjugative lncFII multi-resistance plasmids carrying *bla*_CTX__–__M_, p11011-fosA and p417957-CTXM, were sequenced and analyzed. Both conjugative plasmids were larger than 100 kb and contained three accessory modules, including MDR region. The MDR region of the two plasmids contained many antibiotic resistance genes, including *bla*_CTX__–__M_, *mph (A)*, *dfrA17*, *aadA5*, *sul1*, etc. After transfer, both the transconjugants displayed elevated MICs of the respective antimicrobial agents. A large number of resistance genes clusters in specific regions may contribute to the MDR profile of the strains. The presence of mobile genetic elements at the boundaries can possibly facilitate transfer among *Enterobacteriaceae* through inter-replicon gene transfer. Our study provides beta-lactam resistance profile of bacteria, reveals the prevalence of β-lactamase resistance genes in livestock and poultry breeding environment in Zhejiang Province, and enriches the research on IncFII plasmids containing *bla*_CTX__–__M_.

## Introduction

*Escherichia coli* is the most common conditional pathogen in the culture environment, which can cause varying degrees of infection in livestock and poultry. These *Escherichia coli* strains, which contain a large number of antibiotic resistant genes, accumulate in humans and farmed animals, and are released into the environment via feces. They continue to contaminate the human food environments (e.g., agricultural, aquatic, and livestock products) as well as livestock and poultry breeding environment (Sajjad et al., 2011; Chajȩcka-Wierzchowska et al., 2014; Yano et al., 2014; Blaak et al., 2015; Liao et al., 2015). Finally, multidrug-resistant *E. coli* accumulate in the human body through the food chain (Wellington et al., 2013).

Extended-spectrum β-lactamase (ESBLs) is currently considered one of the world’s major public health problems. In *Enterobacteriaceae*, *E. coli* causes the most infections and is the main species producing ESBL (Bradford, 2001). Many genes are able to produce ESBLs that they can hydrolyze cephalosporins, penicillin, and monocyclic β-lactam antibiotics aztreonam (Kanayama et al., 2010). β-lactamases have been divided into four major classes (A, B, C, and D) according to the amino acid similarity. β-lactamases of classes A, C, and D are serine β-lactamases, whereas those of class B are metallo-β-lactamases (Paterson and Bonomo, 2005). Initially, the most prevalent ESBLs were TEM or SHV variants possessing amino acid substitutions (Sivaraman et al., 2021). *bla*_CTX__–__M_ is one of the most important ESBL genes, which has been found in several conjugative plasmids of *Enterobacteriaceae* (Abera et al., 2016; Abraham et al., 2018). IncF plasmids are usually low copy number plasmids, > 100 kb in size, and typically carry fused replicons with multiple replication initiation genes (Laura et al., 2010). The multi-replicon status is described as a means by which a plasmid with a narrow host range can replicate in a wide host range. Integrons were initially discovered because they are often associated with drug-resistant genes (Stokes and Hall, 1989). Class 1 integrons are the most frequently identified integron classes within clinical environments. This class was the first integron found, which mainly distributed on plasmids of Gram-negative bacteria (Goldstein et al., 2001). *bla*_CTX__–__M_ gene was used as a query for BLAST search in the NCBI nucleotide sequence database, and it was found that it existed mainly in *Enterobacterales*. However, it is still unknown how frequently the gene occurs in the *E. coli* in Zhejiang Province, and the number of related IncFII plasmids containing *bla*_CTX__–__M_ is not very high in the NCBI database. Moreover, the genetic background of *bla*_CTX__–__M_ also needs to be further studied.

In this study, the relative abundance of the known ESBL genes in *E. coli* in livestock and poultry breeding environment was determined. This research provided beta-lactam resistance profile of bacteria isolated from livestock and poultry breeding environment in Zhejiang Province. Our study suggests that resistance to beta-lactam is too high in *E. coli*. Therefore, local farmers can possibly reduce the use of specific beta-lactam antibiotics, making a particular contribution to the reduction of local antibiotic resistance. Furthermore, two IncFII plasmids harboring *bla*_CTX__–__M_ along with other resistance genes were analyzed to clarify the basis for dissemination. This research enriched the study of plasmids containing *bla*_CTX__–__M_ resistance genes in the NCBI database.

## Materials and Methods

### Bacterial Strains

A total of 206 non-duplicate *E. coli* isolates were collected from 6 livestock and poultry farms in Zhejiang Province, China, 2018. A list of collected samples from feces, urine, soil, and surrounding water environment was presented in Supplementary Table 1. Colonies with a green metallic sheen growing on Eosin Methylene Blue Agar plates were initially considered *E. coli* strains. The isolates were identified by VITEK-2 Compact and 16S rRNA gene identification using the universal 16s primers 27F and 1492R (Lane, 1991). EC600 served as the recipient strain in conjugal transfer experiments. EC600 was resistant to rifampicin, but sensitive to ampicillin, carbapenem, quinolones, and sulfonamides.

### Antimicrobial Susceptibility Testing

*E. coli* strains were evaluated based on the size of the inhibition zones and classified as resistant, intermediate, or susceptible according to the CLSI criteria for *Enterobacteriaceae* as the breakpoints were shown in Supplementary Table 2 (Institute CLSI, 2018). *E. coli* strains exhibiting resistance to three or more classes of antimicrobials were considered to be multi-drug resistant (MDR). *E. coli* ATCC 25922 served as the control.

### PCR Analysis

*E. coli* strains were screened for the presence of the ESBLs gene by PCR using the primers listed in Supplementary Table 3 (*bla*_GES_, *bla*_KPC_, *bla*_SME_, *bla*_IMI_, etc.) and Supplementary Table 4 (*bla*_CTX__–__M_ universal, *bla*_CTX__–__M__–__1_, *bla*_CTX__–__M__–__2_, *bla*_CTX__–__M__–__8_, *bla*_CTX__–__M__–__9_, *bla*_CTX__–__M__–__25_, etc.), as described previously (Hong et al., 2012). PCR amplification consisted of denaturation at 95°C for 5 min followed by denaturation at 94°C for 30 s, annealing at their respective annealing temperature for 30 s, and polymerization at 72°C for 40 s for a total of 30 cycles, and a final extension at 72°C for 10 min. All the PCR products were subjected to Sanger sequencing.

### The Enzyme-Producing Activity of Bacteria

The enzyme-producing activity was detected by a modified Carba NP method. The carbapenems producing types of *Enterobacteriaceae* mainly include A, B, and D, representing KPC, NDM, and OXA-48, respectively. According to the characteristics of the above enzymes, an improved Carba NP detection method was designed to detect specific enzyme production types. Class A carbapenems were at least partially inhibited by tazobactam, while class B carbapenems (metal-β-lactamases) were inhibited by divalent cationic chelators such as EDTA. No chemical inhibitors were available for class D carbapenems. The formulas for each system and the expected outcomes of different enzyme production types were shown in Supplementary Table 5. 1705 was the control of enzyme A, and the well of substrate II and substrate IV turned yellow. 2146 was the control of enzyme B, and the wells of substrate II and substrate III turned yellow.

### Conjugal Transfer Experiments

Conjugal transfer experiments were performed with rifampin-resistant *E. coli* EC600 used as a recipient and each of the *bla*_CTX__–__M_-positive 11011 and 417957 isolates as a donor. Brain Heart Infusion (BHI) was supplemented with 2.5 mg/mL rifampicin and 200 μg/L ampicillin to screen transconjugants. After incubation at 37°C for 24 h, colonies growing on these selective plates were further confirmed by antimicrobial sensitivity experiments and VITEK-2 Compact.

### Sequencing and Analysis

The two conjugatable plasmids of the transconjugants 11011-EC600 and 417957-EC600 were sequenced by the Illumina MiSeq platforms. The two plasmids DNA was extracted from the transconjugants 11011-EC600 and 417957-EC600 using the Qiagen Large-Construct Kit. After extraction, the mate-pair library was constructed. Then MiSeq (Illumina, CA, United States) was used to sequence the plasmids, and Newbler 2.6 was used to assemble the Illumina read sets straight off the MiSeq. Quality control and removing low-quality data were performed with TrimMomatic 0.36 (Bolger et al., 2014). Gapfiller V1.11 is used to fill gaps (Nederbragt, 2014). Finally, Cytoscape was used to spline the sequence to obtain the final cyclized plasmids. The plasmid sequences were submitted to Rast, Genemarks, Glimmer, and Prodigal library for preliminary gene prediction then submitted to ISFinder, Integrall, ResFinder, and TN Number Registry for mobile elements (Boetzer and Pirovano, 2012). Gene organization diagrams were drawn by running a gene alignment program in Perl language (Supplementary File) with Ubuntu 18.04 LTS^1^ and Inkscape 0.48.1.^2^ The complement sequences of the two plasmids p417957-CTXM and p11011-fosA have been submitted in GenBank under accession numbers NZ_MH454107.1 and NZ_MG764548.1, respectively.

## Results

### Detection of the bla_CTX__–__M_ Gene in *Escherichia coli* Strains

In this study, 206 strains of *E. coli* were identified by VITEK-2 Compact and the amplification of 16S rRNA gene (Supplementary Table 6). A total of 137 resistant strains to cephalosporins and carbapenems, isolated from farms, were screened for β-lactamase resistance genes (Table 1). The results showed that six extended-spectrum β-Lactamase resistance genes were detected in all antibiotic resistant strains, including *bla*_TEM_, *bla*_CTX__–__M_, *bla*_SHV_, *bla*_OAX__–__1_, *bla*_KPC_, and *bla*_NDM_. The detection rates were 94.89% (130/137), 92.7% (127/137), 24.81% (34/137), 20.43% (28/137), 0.72% (1/137), and 0.72% (1/137), respectively. Only two strains carried the carbapenemases-producing resistance gene. Strain 11011 contained *bla*_KPC_, and strain 417957 contained *bla*_NDM_, which both contained *bla*_CTX__–__M_ antibiotic resistance gene and were selected for further investigation.

**TABLE 1:** Resistant phenotype of 206 *E. coli* from livestock and poultry.

**Antimicrobial agent**	**Susceptible (strain)**	**Susceptible rate**	**Intermediate (strain)**	**Resistant (strain)**	**Resistant rate**
Cefazolin	35	16.99%	34	137	66.50%
Ceftazidime	131	63.59%	0	75	36.40%
Imipenem	201	97.57%	0	5	2.43%

### Determination of the Activity of Carbapenem

The modified Carba NP method was used to detect the type of enzyme production of related test strains. Only two strains out of 206 *E. coli* produced the enzymes. According to the color changes of the test strains that existed in the wells of substrate II, substrate III, and substrate IV, strain 11011 produced enzyme A and strain 417957 produced enzyme B (Supplementary Figure 1).

### The *bla*_CTX__–__M_ Gene Is Transferable

The transconjugants isolated by mating 11011 and 417957 strains with EC600 were resistant to rifampicin and ampicillin antibiotics, likely indicating plasmid transfer. According to the MIC results of wild strains and transconjugant, the resistant plasmid has been successfully introduced into the recipient bacteria (Table 2). The two transconjugants were not resistant to carbapenem but resistant to β-lactam, and both isolates carried two plasmids. It indicated that the plasmid containing *bla*_CTX__–__M_ was conjugatable, and the plasmid containing carbapenem resistance gene was not conjugatable.

**TABLE 2:** The result of MIC by using VITEK-2.

**Category**	**Antimicrobial agent**	**MIC (mg/L)/antimicrobial susceptibility**				
		**11011**	**11011-fosA-EC600**	**417957**	**417957-CTXM-EC600**	**EC600**
Penicillin	Ampicillin	≥32/R	≥ 32/R	≥ 32/R	≥ 32/R	4/S
	Ampicillin/Sulbactam	≥ 32/R	≥ 32/R	≥ 32/R	≥ 32/R	4/S
	Piperacillin	≥ 128/R	≥ 128/R	≥128/R	≥ 128/R	≤ 4/S
	Piperacillin/Tazobactam	≥ 128/R	≥ 128/R	≥ 128/R	≥ 128/R	≤ 4/S
Cephalosporins	Cefazolin	≥ 64/R	≥ 64/R	≥ 64/R	≥ 64/R	≤ 4/S
	Cefuroxime	≥ 64/R	≥ 64/R	≥ 64/R	≥ 64/R	16/I
	Cefuroxime Axetil	≥ 64/R	≥ 64/R	≥ 64/R	≥ 64/R	16/I
	Ceftriaxone	≥ 64/R	≥ 64/R	≥ 64/R	≥ 64/R	≤ 1/S
	Cefepime	≥ 64/R	≥ 64/R	≥ 64/R	≥ 64/R	≤ 1
Monobactams	Aztreonam	≥ 64/R	≥ 64/R	≥ 64/R	≥ 64/R	≤ 1/S
Carbapenems	Imipenem	≥ 16/R	≤ 1/S	≥ 16/R	≤ 1/S	≤ 1/S
	Meropenem	≥ 16/R	≤ 0.25/S	≥ 16/R	≤ 0.25/S	≤ 0.25/S
Aminoglycosides	Amikacin	≤ 2/S	≤ 2/S	≤ 2/S	≤ 2/S	≤ 2/S
	Gentamicin	≤ 1/S	≤ 1/S	≤ 1/S	≤ 1/S	≤ 1/S
	Tobramycin	≤ 1/S	≤ 1/S	≤ 1/S	≤ 1/S	≤ 1/S
Quinolones	Ciprofloxacin	≥ 4/R	≤ 0.25/S	≥ 4/R	≤ 0.25/S	≤ 0.25/S
	Levofloxacin	≥ 8/R	≤ 0.25/S	≥ 8/R	≤ 0.25/S	0.5/S
Nitrofurantoins	Nitrofurantoin	128/R	≤ 16/S	128/R	≤ 16/S	≤ 16S
Sulfonamides	Trimethoprim/Sulfamethoxazole	≥ 320/R	≤20/S	≥ 320/R	≤ 20/S	≤ 20/S

*S, Susceptible; R, Resistant; I, Intermediate.*

### Characterization of Plasmids p11011-fosA and p417957-CTXM

**FIGURE 1:**
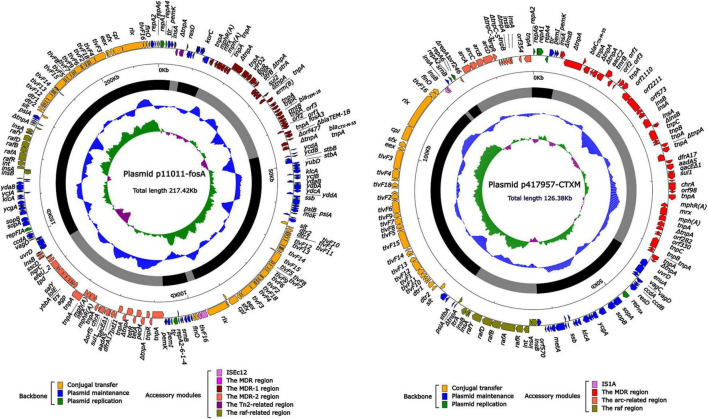
The complete sequence diagram of p11011-fosA and p417957-CTXM. Note: In the figure, the innermost ring represents (G-C)/(G + C); the blue ring indicates GC content, and the outward depression indicates that the GC content is higher than the mean value. Black area in the outer circle represents the backbone area and gray area represents the accessory modules. The outermost circle shows the distribution of genes represented by colored arrows in the plasmid.

As shown in Table 3 and Figure 1, the total length of plasmid p11011-fosA was 217.4 kb, contained 275 ORFs in total. The content of GC was 52.7%, and the plasmid contained three accessory modules, comprising Tn*2*-related region, MDR-1 region, and *raf*-related region. The total length of plasmid p417957-CTXM was 126.4 kb, which included 151ORFs. GC content was 51.4%, and the plasmid also contained three accessory modules, comprising MDR, *raf*- and *arc*-related regions. The plasmid backbone sequences of the two plasmids were similar to that of plasmid pHN7A8 from *Klebsiella pneumoniae.* The single replicon IncFII plasmid pHN7A8 (Genbank: NC_019073.1) was chosen as the reference plasmid. The length of plasmid pHN7A8 was 76.8 kb, and only contained one accessory modules-a 14.5 kb MDR region. The three plasmids consist of the backbone area and accessory modules. They were formed by inserting antibiotic resistance genes and adjacent mobile elements into different positions in the backbone area. Both plasmids p11011-fosA and p417957-CTXM contain numerous antibiotic resistance genes (Table 4), and plasmids can be transferred to EC600 by conjugation.

**TABLE 3:** Major features of plasmids analyzed.

**Type**	**Plasmid Name**		
	**p11011-fosA**	**p417957-CTXM**	**pHN7A8**
Sequence Type	*[F36:A4:B-]*	*[F22:A1:B-]*	*[F33:A-:B-]*
Allele	FIA_4, FII_36, FII_33	FIA_1, FII_22, FII_36	FII_33
The total length(kb)	217.4	126.4	76.8
The number of ORF	275	151	102
Average GC content (%)	53.92	53.82	54.52
Accessory modules	Tn*2*-related region, MDR-1 region and *raf*-related region	MDR region, *raf*-region and *arc*-related region	MDR region

**TABLE 4:** Antibiotic resistance genes in sequenced plasmids.

**Plasmid**	**Resistance gene**	**Resistance phenotype**	**Nucleotide position**	**Region located**
p11011-fosA	*mph(A)*	Macrolides resistance	17065.17970	MDR-1 region
	*floR*	Florfenicol resistance	25301.26515	
	*strAB*	Streptomycin resistance	27471.29110	
	*sul2*	Sulfonamides resistance	29171.29986	
	*erm(B)*	Macrolides resistance	31936.32673	
	*bla*_TEM__–1B_	β-lactam resistance	36041.36091	
	*fosA3*	Fosfomycin resistance	40467.40883	
	*bla*_CTX__–__M__–__55_	β-lactam resistance	43548.44423	
	*tetAR*	Tetracycline resistance	113327.115182	MDR-2 region
	*dfrA17*	Trimethoprim resistance	119457.119930	
	*aadA5*	Aminoglycosides resistance	120016.120849	
	*sul1*	Sulfonamides resistance	121309.122235	
	*mph(A)*	Macrolides resistance	127546.128451	
p417957-CTXM	*bla*_CTX__–__M__–__55_	β-lactam resistance	7839.8714	MDR-1 region
	*aacC2*	Aminoglycosides resistance	11222.12083	
	*tmrB*	Tunicamycin resistance	12095.12637	
	*dfrA17*	Trimethoprim resistance	27180.27653	
	*aadA5*	Aminoglycosides resistance	27784.28572	
	*sul1*	Sulfonamides resistance	29032.29958	
	*mph(A)*	Macrolides resistance	35269.36174	

### Replicon repA2-6-1-4 of the Backbone Area

**FIGURE 2:**
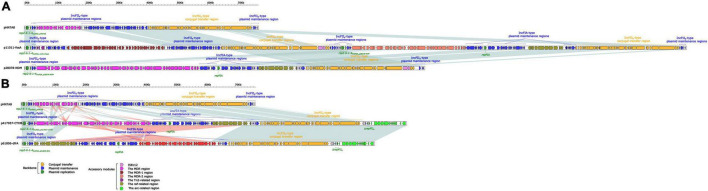
**(A)** Linear comparison of p11011-fosA with related plasmids. **(B)** Linear comparison of p417957-CTXM with related plasmids. Genes are indicated by arrows. Genes, mobile elements and other features are colored based on functional classification. Shadows indicate nucleotide homology is greater than 95%. p11011-fosA and p417957-CTXM were sequenced in this study, while the sequence of pHN7A8, p28078-NDM, and p61806-*dfrA* was from GenBank as reference plasmids.

Comparing the sequences in the database revealed that both plasmids p11011-fosA and p417957-CTXM belonged to multi-replicon IncFII plasmids, which both contained the main replicon repA2-6-1-4 (Figure 2). The sequence of this gene was identical to the replication initiation region of the reference plasmid pHN7A8. p417957-CTXM also contained a repFIA replicon and the backbone area, including core butler genes (*ccdAB*, *vagCD*, *sopAB*, *ycgA*, *klcA*, and *ssb*) responsible for maintaining the stability of the plasmid. In addition to the main replicon repA2-6-1-4 and the auxiliary replicon repFIA, plasmid p11011-fosA also contained a replicon repA2-6-1-4 structure. The sequence of this structure was different from that of pHN7A8. Therefore, the plasmid was likely to be hybridized by two IncFII plasmids. The backbone area of p417957-CTXM had a conjugal transfer region similar to the reference plasmid pHN7A8, considering their nucleic acid alignment and expressed proteins. The plasmid p11011-fosA contained two conjugal transfer regions. Conjugal transfer proteins and flagella proteins promoted flagella production, which collectively facilitated horizontal transfer of plasmids between bacteria.

### Analysis of Multi-Drug Resistant Region in p11011-fosA and p417957-CTXM

**FIGURE 3:**
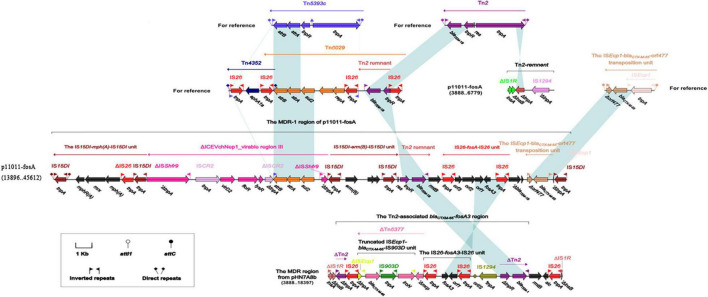
The MDR-1 region of p11011-fosA. Genes are indicated by arrows. Genes, mobile elements and other features are colored based on functional classification. Shadows indicate nucleotide homology is greater than 95%. The number in parentheses indicates the location of the fragment in the plasmid.

As shown in Figure 3, the MDR-1 region of plasmid p11011-fosA consists of the following mobile elements: IS*15DI*-*mph (A)*-IS*15DI* unit [containing macrolide resistance gene *mph (A)*], ΔICEVchNep1_virable region III (containing florfenicol resistance gene *floR*, streptomycin resistance gene *strAB*, sulfonamides resistance gene *sul2*), IS*15DI*-*erm (B)*-IS*15DI* unit [macrolide resistance genes *erm (B)*], Tn*2* remnant, IS*26*-*fosA*-IS*26* unit (containing fosfomycin resistance gene *fosA3*), and IS*Ecp1*-*bla*_CTX__–__M__–__55_-*orf477* transposition unit (containing β-lactam antibiotic *bla*_CTX__–__M__–__55_). The MDR region of pHN7A8 was similar to the MDR-1 region of p11011-fosA, which contained fosfomycin resistance genes mediated by IS*26* and aminoglycoside resistance genes mediated by Tn*2*.

The MDR region of p417957-CTXM contained integron In*54*, IS*26*-*mph (A)*-*mrx*-*mphR (A)*-IS*6100* unit, IS*Ecp1*-*bla*_CTX__–__M__–__55_-*orf477* transposition unit (containing β-lactamase resistance gene *bla*_CTX__–__M__–__55_), and *aacC2*-*tmrB* region (containing aminoglycoside resistance gene *aacC2* and tunicamycin resistance gene *tmrB*).

**FIGURE 4:**
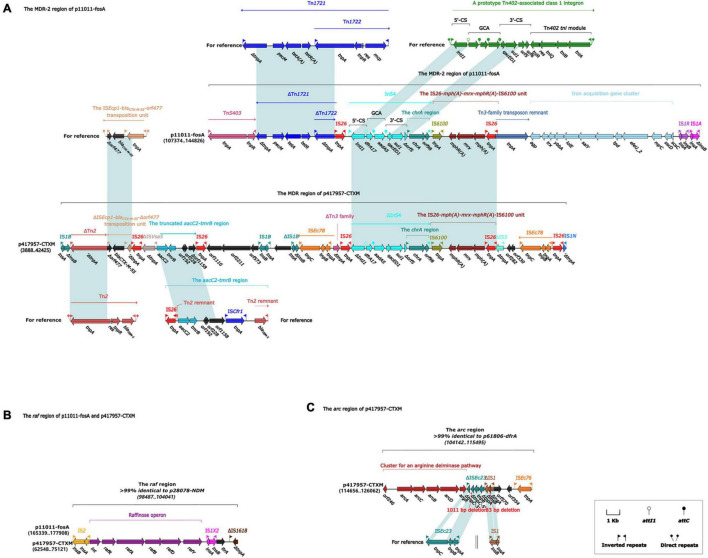
**(A)** The MDR-1 region of p11011-fosA and MDR region of p417957-CTXM. **(B)** The *raf* region of p110011-*fosA* and p417957-CTXM. **(C)** The *arc* region of p417957-CTXM. Genes are indicated by arrows. Genes, mobile elements, and other features are colored based on functional classification. Shadows indicate nucleotide homology is greater than 95%. The number in parentheses indicates the location of the fragment in the plasmid.

The multi-drug resistance region MDR-2 of the plasmid p11011-fosA was composed of the following mobile elements in sequence: Tn*5403*, ΔTn*1721* (containing tetracycline resistance gene *tetAR*), IS*26*, In*54* (containing trimethoprim resistance gene *drfA17*, sulfonamides resistance gene *sul1*), IS*26*-*mph(A)*-*mrx*-*mphR (A)*-IS*6100* unit (containing macrolide resistance genes), Tn*3* family transposon remnant, Iron acquisition gene cluster, IS*1R* and IS*1A* (Figure 4). The typical structure of type 1 integron included IRi, 5 prime -CS, GCA, 3 prime -CS, Tn*402* tni module, and IRt. In*54* was a type I integron in both plasmids p11011-fosA and p417957-CTXM. The 5 prime -CS region contained integrase *Intl1* responsible for the integration of capture resistance genes, and downstream of *Intl1* contained trimethoprim resistance gene *dfrA17* and aminoglycoside resistance gene *aadA5*. In addition, the 3 prime -CS region also contained sulfonamides resistance gene *sul1*. Moreover, the *tni* region of In*54* was replaced by the *chrA* region, which was responsible for chromate particle transport. *ChrA* region was usually located in IS*26*-*mph (A)*-*mrx*-*mphR (A)*-IS*6100* unit.

## Discussion

In this study, *E. coli* strains isolated from the livestock and poultry breeding environment in Zhejiang Province showed high-level resistance (137/204) to extended-spectrum β-lactam antibiotics (Cefazolin, Ceftazidime, and Imipenem). These antibiotics are prevalently used for the treatment of infections caused by *Enterobacteriaceae*. Essentially, all ESBL producers demonstrated multi-drug resistance profiles and carried at least one ESBL resistance gene, especially *bla*_TEM_ (94.89%) and *bla*_CTX__–__M_ (92.7%). Among all the *E. coli* strains investigated in the current study, the prevalence of ESBL producers (67.2%) is quite close to that previously reported in Henan Province (60.8%) from 2007 to 2008 (Yuan et al., 2009). However, it is still lower than those reported in northeast China (Jilin, Liaoning, and Heilongjiang Provinces (100%) from 2011 to 2013 (Tong et al., 2015).

In this study, six extended-spectrum β-Lactamase resistance genes were detected in all antibiotic resistant strains, and *bla*_CTX__–__M_ was the second common resistance gene. However, Tong et al. (2015) found that CTX-M-type ESBL resistance genes were the most common genotype of *E. coli* isolated from chickens in China. These contradictories may be due to different regions where the strains were isolated and various poultry species. However, it might be related to mobile genetic elements such as plasmids and transposons as well. Previous reports showed that CTX-M-encoding genes in *E. coli*, isolated from food animals, have a high diversity in China, and CTX-M-14 was the most widespread CTX-M enzyme, followed by CTX-M-55 (Zheng et al., 2012). *bla*_CTX__–__M__–__55_ has been widely reported in food-producing animals and pets in China (Rao et al., 2014). In this study, the strains carrying CTX-M-55-type ESBL showed different MDR profiles. In order to further study the mobile genetic elements related to CTX-M gene transfer, we selected two plasmids of two strains for sequencing and analysis.

We identified two conjunctive IncFII multi-resistance plasmids carrying *bla*_CTX__–__M__–__55_ and analyzed the genetic context of *bla*_CTX__–__M__–__55_ and MDR region. Most CTX-M genes were identified to be associated with IncFII or IncI1 plasmids (Carattoli, 2009; Cao et al., 2011). The most common IncFII type plasmid was F2:A-: B-, which had also been reported to be linked to *bla*_CTX__–__M_ genes in *Enterobacteriaceae* strains isolated from other regions (Laura et al., 2010; Kim et al., 2011). In the current study, the IncFII plasmid p11011-fosA contains another ESBL resistance genes *bla*_TEM__–1B_ and fosfomycin resistance gene *fosA3*, while the other IncFII plasmid p417957-CTXM don’t have the two resistance genes. *fosA3* is frequently co-transferred with *bla*_CTX__–__M_ genes, and the dissemination of *fosA3* may be related to the co-selection of cephalosporins (Hou et al., 2012). In previous epidemic plasmids, it was observed that the *fosA3* gene is frequently co-transferred with *bla*_CTX__–__M__–__14_, *bla*_CTX__–__M__–__55_, *bla*_CTX__–__M__–__65_, *floR*, and *rmtB* genes. Thus, reducing the total use of antibiotics, especially cephalosporins, in livestock and poultry breeding environment contributes to controlling the dissemination of plasmid-mediated fosfomycin and β-Lactamase resistance genes (Yang et al., 2014).

In this study, we found that *bla*_CTX__–__M__–__55_ was located on the IS*Ecp1*-*bla*_CTX__–__M__–__55_-*orf477* transposition unit, which was similar to previous studies (Zheng et al., 2012). IS*Ecp1*-*bla*_CTX__–__M__–__55_-*orf477* transposition unit is a significant element in the propagation of *bla*_CTX__–__M__–__55_. IS*Ecp1* is bounded by 14-bp inverted repeats, with IR_L_ at the left end of the transcription direction of the transposase gene and IR_*R*_ at the right end. In addition, IS*Ecp1* uses IR_L_ in conjunction with alternative sequences like these IR to mobilize adjacent regions, forming a 5-bp direct repeat (DR) on transposition (Poirel et al., 2005; Wachino et al., 2006). The MDR region in pHN7A8 and p11011-fosA is mainly formed by the insertion of mobile elements. The insertion of Tn*2*-*rmtB* element into the i*nsB* of IS*1R* results in only 214 bp residue in *insB*. The insertion of the region of 9.4 kb [IS*26*, Δ Tn*6377*, the IS*26*-*fosA3*-IS*26* unit (Ho et al., 2013) and IS*1294*] between *tnpA* and *tnpR* of Tn*2* leads to the loss of *res* site of Tn*2*. Tn*2*-*rmtB* element (Doi et al., 2008) and the variant of this element have been widely found in *Enterobacteriaceae*. Tn*6377* was originally identified on the plasmid pKP96 (Shen et al., 2008) in *Klebsiella pneumoniae*, and the transposon was formed by inserting Tn*1722* (Zong et al., 2011) into the IS*Ecp1* – *bla*_CTX__–__M__–__65_-IS *903D* unit of the Tn*3* family. Tn*6377* in pHN7A8 is interrupted at both terminals by IS*26*.

The MGEs, such as transposons, play an essential role in the rapid spread of AMR by horizontal gene transfer due to the selective pressure imposed by antibiotics (Snesrud et al., 2018). Tn*5403* found in MDR-2 region in plasmid p11011-fosA did not contain antibiotic resistance genes and was first identified in the plasmid of *Klebsiella pneumoniae* (Rinkel et al., 1994). Another transposon Tn*1721* belongs to the transposon of the Tn*21* family and has three 38 bp repeats and two transposase genes *tnpA* with the same sequence. There are *tnpA*, *tnpR*, *res* sites, and the gene *mcp* that encodes methyl-chemotactic proteins at one terminal, with 38 bp reverse repeats on both sides of this terminal. This terminal is named Tn*1722* and can be moved independently. The rest of Tn*1721* contains the tetracycline resistance gene *tetA*. Although Tn*1721* in the MDR-2 region of p11011-fosA is interrupted by IS*26* and Tn*5403*, the key tetracycline resistance gene in this transposon remains intact. IS*26*-*mph (A)*-*mrx*-*mphR (A)*-IS*6100* unit is the carrier element for common macrolide resistance genes, which are usually located on resistant plasmids in *E. coli* (Partridge, 2011).

## Conclusion

The presence of multiple mobile elements in a *bla*_CTX__–__M_-carrying multi-resistance plasmid makes it flexible. These elements aid its persistence and dissemination among *E. coli* and potentially other Gram-negative bacteria. This study demonstrated the abundance of β-lactamase resistance genes in livestock and poultry breeding environment in Zhejiang Province. It also upgraded the research on IncFII plasmids containing *bla*_CTX__–__M_ antibiotic resistance genes in the NCBI database. This research also provided beta-lactam resistance profile of bacteria isolated from farms in Zhejiang Province, indicating that local farmers can possibly reduce the use of specific beta-lactam antibiotics to reduce the evolution of multi-resistant strains.

## Data Availability Statement

Publicly available datasets were analyzed in this study. This data can be found here: https://www.ncbi.nlm.nih.gov/nuccore/NZ_MH454107.1 and https://www.ncbi.nlm.nih.gov/nuccore/NZ_MG764548. 1NCBI, GenBank accession numbers NZ_MH454107.1 and NZ_MG764548.1.

## Author Contributions

WZ: visualization, data curation, software, and writing—original draft preparation. EZ: conceptualization, methodology, formal analysis, and writing—original draft preparation. JZ: visualization and investigation. ZH: software and writing—review and Editing. YZ: software and validation. JH: formal analysis and resources. DQ: conceptualization, resources, supervision, and writing—review and Editing. All authors contributed to the article and approved the submitted version.

## Conflict of Interest

The authors declare that the research was conducted in the absence of any commercial or financial relationships that could be construed as a potential conflict of interest.

## Publisher’s Note

All claims expressed in this article are solely those of the authors and do not necessarily represent those of their affiliated organizations, or those of the publisher, the editors and the reviewers. Any product that may be evaluated in this article, or claim that may be made by its manufacturer, is not guaranteed or endorsed by the publisher.

## Footnotes

1
https://ubuntu.com/

2
https://inkscape.org/en/

## References

[B1] Abera B. Kibret M. Mulu W. (2016). Extended-Spectrum beta (β)-lactamases and antibiogram in *Enterobacteriaceae* from clinical and drinking water sources from bahir dar city, Ethiopia. *PLoS One* 11:e0166519. 10.1371/journal.pone.0166519 27846254 PMC5112950

[B2] Abraham S. Kirkwood R. N. Laird T. Saputra S. Mitchell T. Singh M. (2018). Dissemination and persistence of extended-spectrum cephalosporin-resistance encoding IncI1-*bla*(_*CTXM–1*_) plasmid among *Escherichia coli* in pigs. *ISME J.* 12 2352–2362. 10.1038/s41396-018-0200-3 29899511 PMC6155088

[B3] Blaak H. Lynch G. Italiaander R. Hamidjaja R. A. Schets F. M. de Roda Husman A. M. (2015). Multidrug-Resistant and extended spectrum beta-lactamase-producing *Escherichia coli* in dutch surface water and wastewater. *PLoS One* 10:e0127752. 10.1371/journal.pone.0127752 26030904 PMC4452230

[B4] Boetzer M. Pirovano W. (2012). Toward almost closed genomes with GapFiller. *Genome Biol.* 13:R56. 10.1186/gb-2012-13-6-r56 22731987 PMC3446322

[B5] Bolger A. M. Lohse M. Usadel B. (2014). Trimmomatic: a flexible trimmer for Illumina sequence data. *Bioinformatics* 30 2114–2120. 24695404 10.1093/bioinformatics/btu170 PMC4103590

[B6] Bradford P. A. (2001). Extended-spectrum beta-lactamases in the 21st century: characterization, epidemiology, and detection of this important resistance threat. *Clin. Microbiol. Rev.* 14:933. 10.1128/CMR.14.4.933-951.2001 11585791 PMC89009

[B7] Cao X. Cavaco L. M. Lv Y. Li Y. Zheng B. Wang P. (2011). Molecular characterization and antimicrobial susceptibility testing of *Escherichia coli* isolates from patients with urinary tract infections in 20 Chinese hospitals. *J. Clin. Microbiol.* 49 2496–2501. 10.1128/jcm.02503-10 21525216 PMC3147837

[B8] Carattoli A. (2009). Resistance plasmid families in *Enterobacteriaceae*. *Antimicrob. Agents Chemother.* 53 2227–2238. 10.1128/aac.01707-08 19307361 PMC2687249

[B9] Chajȩcka-Wierzchowska W. Zadernowska A. Nalepa B. Sierpińska M. Laniewska-Trokenheim L. (2014). Retail ready-to-eat food as a potential vehicle for *Staphylococcus spp.* harboring antibiotic resistance genes. *J. Food Prot.* 77 993–998. 10.4315/0362-028x.Jfp-13-466 24853524

[B10] Doi Y. Adams-Haduch J. M. Paterson D. L. (2008). *Escherichia coli* Isolate Coproducing 16S rRNA Methylase and CTX-M-Type Extended-Spectrum β-Lactamase Isolated from an Outpatient in the United States. *Antimicrob. Agents Chemother.* 52 1204–1205. 10.1128/AAC.01320-07 18195064 PMC2258542

[B11] Goldstein C. Lee M. Sanchez S. Hudson C. Phillips B. Register B. (2001). Incidence of class 1 and 2 integrases in clinical and commensal bacteria from livestock, companion animals, and exotics. *Antimicrob. Agents Chemother.* 45 723–726. 10.1128/AAC.45.3.723-726.2001 11181350 PMC90363

[B12] Ho P. L. Chan J. Lo W. U. Law P. Y. Li Z. Lai E. L. (2013). Dissemination of plasmid-mediated fosfomycin resistance *fosA3* among multidrug-resistant *Escherichia coli* from livestock and other animals. *J. Appl. Microbiol.* 114 695–702. 10.1111/jam.12099 23216653

[B13] Hong S. S. Kyeongmi K. Young H. J. Bochan J. Kang M. S. Hong S. G. (2012). Multiplex PCR for rapid detection of genes encoding Class A Carbapenemases. *Ann. Lab. Med.* 32 359–361. 10.3343/alm.2012.32.5.359 22950072 PMC3427824

[B14] Hou J. Huang X. Deng Y. He L. Yang T. Zeng Z. (2012). Dissemination of the fosfomycin resistance gene *fosA3* with CTX-M β-lactamase genes and *rmtB* carried on IncFII plasmids among *Escherichia coli* isolates from pets in China. *Antimicrob. Agents Chemother.* 56 2135–2138. 10.1128/aac.05104-11 22232290 PMC3318358

[B15] Institute CLSI (2018). *Performance Standards for Antimicrobial Susceptibility Testing—Twenty-Eighth Edition: M100”.* Wayne, PA: CLSI.

[B16] Kanayama A. Iyoda T. Matsuzaki K. Saika T. Ikeda F. Ishii Y. (2010). Rapidly spreading CTX-M-type beta-lactamase-producing *Proteus mirabilis* in Japan. *Int. J. Antimicrob. Agents* 36 340–342. 10.1016/j.ijantimicag.2010.06.002 20609568

[B17] Kim J. Bae I. K. Jeong S. H. Chang C. L. Lee C. H. Lee K. (2011). Characterization of IncF plasmids carrying the *bla*_*CTX–M–14*_ gene in clinical isolates of *Escherichia coli* from Korea. *J. Antimicrob. Chemother.* 66 1263–1268. 21415040 10.1093/jac/dkr106

[B18] Lane D. J. (1991). “16S/23S rRNA sequencing,” in *Nucleic Acid Techniques in Bacterial Systematics*, eds Stackebrandt E. Goodfellow M. (New York, NY: John Wiley and Sons).

[B19] Laura V. Aurora G. F. Daniela F. Alessandra C. (2010). Replicon sequence typing of IncF plasmids carrying virulence and resistance determinants. *J. Antimicrob. Chemother.* 65 2518–2529. 10.1093/jac/dkq347 20935300

[B20] Liao X. P. Xia J. Yang L. Li L. Sun J. Liu Y. H. (2015). Characterization of CTX-M-14-producing *Escherichia coli* from food-producing animals. *Front. Microbiol.* 6:1136. 10.3389/fmicb.2015.01136 26528278 PMC4606122

[B21] Nederbragt A. J. (2014). On the middle ground between open source and commercial software-the case of the Newbler program. *Genome Biol.* 15 1–2. 10.1186/gb4173 25180324 PMC4054848

[B22] Partridge S. R. (2011). Analysis of antibiotic resistance regions in Gram-negative bacteria. *Fems Microbiol. Rev.* 35 820–855. 21564142 10.1111/j.1574-6976.2011.00277.x

[B23] Paterson D. L. Bonomo R. A. (2005). Extended-spectrum beta-lactamases: a clinical update. *Clin. Microbiol. Rev.* 18 657–686. 10.1128/cmr.18.4.657-686.2005 16223952 PMC1265908

[B24] Poirel L. Lartigue M. F. Decousser J. W. Nordmann P. (2005). ISEcp1B-mediated transposition of *bla*_*CTX–M*_ in *Escherichia coli*. *Antimicrob. Agents Chemother.* 49 447–450. 10.1128/aac.49.1.447-450.2005 15616333 PMC538921

[B25] Rao L. Lv L. Zeng Z. Chen S. He D. Chen X. (2014). Increasing prevalence of extended-spectrum cephalosporin-resistant *Escherichia coli* in food animals and the diversity of CTX-M genotypes during 2003-2012. *Vet. Microbiol.* 172 534–541. 10.1016/j.vetmic.2014.06.013 24999233

[B26] Rinkel M. Hubert J. C. Roux B. Lett M. C. (1994). Identification of a new transposon Tn*5403* in a *Klebsiella pneumoniae* strain isolated from a polluted aquatic environment. *Curr. Microbiol.* 29:249. 7765419 10.1007/BF01577436

[B27] Sajjad A. Holley M. P. Labbate M. Stokes H. W. Gillings M. R. (2011). Preclinical class 1 integron with a complete Tn*402*-like transposition module. *Appl. Environ. Microbiol.* 77 335–337. 10.1128/aem.02142-10 21037292 PMC3019745

[B28] Shen P. Jiang Y. Zhou Z. Zhang J. Yu Y. Li L. (2008). Complete nucleotide sequence of pKP96, a 67 850 bp multiresistance plasmid encoding *qnrA1*, *aac(6’)-Ib-cr* and *bla*_*CTX–M–24*_ from *Klebsiella pneumoniae*. *J. Antimicrob. Chemother.* 62 1252–1256. 10.1093/jac/dkn397 18812424

[B29] Sivaraman G. K. Rajan V. Vijayan A. Elangovan R. Prendiville A. Bachmann T. T. (2021). Antibiotic resistance profiles and molecular characteristics of extended-spectrum beta-lactamase (ESBL)-producing *Escherichia coli* and *Klebsiella pneumoniae* isolated from shrimp aquaculture farms in Kerala, India. *Front. Microbiol.* 12:622891. 10.3389/fmicb.2021.622891 34489875 PMC8417373

[B30] Snesrud E. Mcgann P. Chandler M. (2018). The birth and demise of the is *Apl1* - *mcr-1* -IS *Apl1* composite transposon: the vehicle for transferable colistin resistance. *mBio* 9 e2381–e2417. 10.1128/mBio.02381-17 29440577 PMC5821093

[B31] Stokes H. W. Hall R. M. (1989). A novel family of potentially mobile DNA elements encoding site-specific gene-integration functions: integrons. *Mol. Microbiol.* 3 1669–1683. 10.1111/j.1365-2958.1989.tb00153.x 2560119

[B32] Tong P. Sun Y. Ji X. Du X. Guo X. Liu J. (2015). Characterization of antimicrobial resistance and extended-spectrum β-lactamase genes in *Escherichia coli* isolated from chickens. *Foodborne Pathog. Dis.* 12:345. 25785885 10.1089/fpd.2014.1857

[B33] Wachino J. Yamane K. Kimura K. Shibata N. Suzuki S. Ike Y. (2006). Mode of transposition and expression of 16S rRNA methyltransferase gene *rmtC* accompanied by IS*Ecp1*. *Antimicrob. Agents Chemother.* 50 3212–3215. 10.1128/aac.00550-06 16940134 PMC1563517

[B34] Wellington E. M. Boxall A. B. Cross P. Feil E. J. Gaze W. H. Hawkey P. M. (2013). The role of the natural environment in the emergence of antibiotic resistance in gram-negative bacteria. *Lancet Infect. Dis.* 13 155–165. 10.1016/s1473-3099(12)70317-1 23347633

[B35] Yang X. Liu W. Liu Y. Wang J. Lv L. Chen X. (2014). F33: A-: B-, IncHI2/ST3, and IncI1/ST71 plasmids drive the dissemination of *fosA3* and *bla*_*CTX–M–55/–14/–65*_ in *Escherichia coli* from chickens in China. *Front. Microbiol.* 5:688. 10.3389/fmicb.2014.00688 25566207 PMC4267423

[B36] Yano Y. Hamano K. Satomi M. Tsutsui I. Ban M. Aueumneoy D. (2014). Prevalence and antimicrobial susceptibility of *Vibrio* species related to food safety isolated from shrimp cultured at inland ponds in Thailand. *Food Control* 38 30–36.

[B37] Yuan L. Liu J. H. Hu G. Z. Pan Y. S. Liu Z. M. Mo J. (2009). Molecular characterization of extended-spectrum beta-lactamase-producing *Escherichia coli* isolates from chickens in Henan Province, China. *J. Med. Microbiol.* 58 1449–1453. 10.1099/jmm.0.012229-0 19574412

[B38] Zheng H. Zeng Z. Sheng C. Liu Y. Liu J. H. (2012). Prevalence and characterisation of CTX-M β-lactamases amongst *Escherichia coli* isolates from healthy food animals in China. *Int. J. Antimicrob. Agents* 39 305–310. 22325120 10.1016/j.ijantimicag.2011.12.001

[B39] Zong Z. Yu R. Wang X. Lu X. (2011). bla_*CTX–M–65*_ is carried by a Tn*1722*-like element on an IncN conjugative plasmid of ST131 *Escherichia coli*. *J. Med. Microbiol.* 60:435–441. 21163826 10.1099/jmm.0.026997-0

