# Supplementary material for: Characterization and Comparative Genomics Analysis of lncFII Multi-Resistance Plasmids Carrying blaCTX–M and Type1 Integrons From Escherichia coli
Source: Front Microbiol. 2021 Nov 16;12:753979. doi: 10.3389/fmicb.2021.753979 (PMC8637017; doi:10.3389/fmicb.2021.753979)
Supplement: Supplementary file 1 [file Data_Sheet_1.zip › Supplementary File 1.DOCX]

#!/usr/bin/perl -w

#========================================================================

# Author: Lizhi.Xu

# Email: xulzh@hotmail.com

#========================================================================

use strict;

use warnings;

use FindBin qw($Bin $Script);

#use SVG;

#use lib "$FindBin::Bin/lib";

use Getopt::Long;

my $VERSION = "0.2.0";

my %opts = (title => 'Plasmid Name', window => 2000, step => 100, radius => 500, mingene => 300, color => "$Bin/color.cfg");

GetOptions(\%opts, 'output=s', 'window=i', 'step=i', 'color=s', 'radius=i', 'mingene=i', 'title=s', 'frame=s', 'desc=s', 'unname', 'help') or &usage(2, $!);

# check options

&usage(2, "Show the help message\n") if (exists $opts{help});

&usage(1, "Input was not accepted, use '-help' to get more message\n") if (@ARGV != 2);

#die "failed to open file: $Bin/display_color.list\n" unless (-e "$Bin/display_color.list");

#default use color list

my %COLOR = ('GC_Skew+' => 'Green', 'GC_Skew-' => 'Purple', 'GC_Content' => 'Blue');

my %FUN;

open (FH, $opts{color}) or die "error: failed to open $opts{color}\n";

while (<FH>) {

chomp;

next if (/^\s*#/ or /^\s*$/);

my ($key, $color) = split (/\t/, $_);

$FUN{$key} = $color;

}

close FH;

my ($seq_file, $list_file) = @ARGV;

#creat svg

my ($seq_id, $desc_str, $seq_str, $size, $gc_mean, @gc_list, @skew_list, @local_list);

open (SEQ, $seq_file) or die $!;

$/ = "\n>";

while (<SEQ>) {

chomp;

next if (/^\s*$/ or /^\s*#/);

my ($head, $seq) = split (/\n+/, $_, 2);

($seq_id, $desc_str) = split (/\s+/, $head, 2);

$seq =~ s/\s+//g;

$size = length $seq;

$seq_str = $seq . substr($seq, 0, $opts{window});

($gc_mean,) = &gc_skew($seq);

}

$/ = "\n";

close SEQ;

my $svg = &new(2*$opts{radius}, 2*$opts{radius}+300);

my $scale = 2*3.1416/$size;

## 中央绘制说明信息，包括标题和长度信息

$svg .= &text($opts{radius}-110, $opts{radius}-20, $opts{title}, stroke =>'black', fill=>'black', 'font-size' => 24) if (defined $opts{title});

$svg .= &text($opts{radius}-80, $opts{radius}+30, sprintf ("Total length %.2fKb", $size/1000), stroke =>'black', fill=>'black', 'font-size' => 18);

## 绘制最小值

#$svg .= &circle($opts{radius}, $opts{radius}, $r-50, stroke => 'gray', fill => 'none');

# count GC

my ($zoom_skew, $zoom_gc) = (0, 0);

for (my $i = $opts{window}; $i < $size+$opts{window}; $i += $opts{step}) {

my ($gc, $skew) = gc_skew(substr($seq_str, $i-$opts{window}, $opts{window}*2));

$zoom_gc = abs($gc-$gc_mean) if (abs($gc-$gc_mean) > $zoom_gc);

$zoom_skew = abs($skew) if (abs ($skew) > $zoom_skew);

push (@local_list, $i*$scale);

push(@gc_list, $gc-$gc_mean);

push(@skew_list, $skew);

}

#warn "## $zoom_skew, $zoom_gc\n";

($zoom_skew, $zoom_gc) = (40/$zoom_skew, 40/$zoom_gc);

#warn "## $zoom_skew, $zoom_gc\n";

my $r = 160;

## 绘制GC－skew

for (my $i = 0; $i < @local_list; $i ++) {

if ($skew_list[$i] > 0) {

$svg .= line($opts{radius}+$r*cos(3.1416/2-$local_list[$i]), $opts{radius}-$r*sin(3.1416/2-$local_list[$i]), $opts{radius}+($r+$skew_list[$i]*$zoom_skew)*cos(3.1416/2-$local_list[$i]), $opts{radius}-($r+$skew_list[$i]*$zoom_skew)*sin(3.1416/2-$local_list[$i]), stroke=>$COLOR{'GC_Skew+'}, fill=>$COLOR{'GC_Skew+'});

} else {

$svg .= line($opts{radius}+$r*cos(3.1416/2-$local_list[$i]), $opts{radius}-$r*sin(3.1416/2-$local_list[$i]), $opts{radius}+($r+$skew_list[$i]*$zoom_skew)*cos(3.1416/2-$local_list[$i]), $opts{radius}-($r+$skew_list[$i]*$zoom_skew)*sin(3.1416/2-$local_list[$i]), stroke=>$COLOR{'GC_Skew-'}, fill=>$COLOR{'GC_Skew-'});

}

}

$r += 80;

## 绘制平均GC含量

for (my $i = 0; $i < @local_list; $i ++) {

$svg .= line($opts{radius}+$r*cos(3.1416/2-$local_list[$i]), $opts{radius}-$r*sin(3.1416/2-$local_list[$i]), $opts{radius}+($r+$gc_list[$i]*$zoom_gc)*cos(3.1416/2-$local_list[$i]), $opts{radius}-($r+$gc_list[$i]*$zoom_gc)*sin(3.1416/2-$local_list[$i]), stroke=>$COLOR{'GC_Content'}, fill=>$COLOR{'GC_Content'});

}

if (exists $opts{frame}) {

$r += 80;

open (INF, $opts{frame}) or die "failed to open file $opts{frame}\n";

while (<INF>) {

chomp;

next if (/^\s*$/ or /^\s*#/);

my ($seq_id, $start, $end, $strand, $len, $type, $color) = split (/\t/, $_);

my @paths;

if ($len < $size/2) {

push (@paths, sprintf ("M%f %f", $opts{radius}+$r*cos(3.1416/2-$start*$scale), $opts{radius}-$r*sin(3.1416/2-$start*$scale)));

push (@paths, sprintf ("A%f %f %f %d %d %f %f", $r, $r, $len*$scale, 0, 1, $opts{radius}+$r*cos(3.1416/2-$end*$scale), $opts{radius}-$r*sin(3.1416/2-$end*$scale)));

push (@paths, sprintf ("L%f %f", $opts{radius}+($r-30)*cos(3.1416/2-$end*$scale), $opts{radius}-($r-30)*sin(3.1416/2-$end*$scale)));

push (@paths, sprintf ("A%f %f %f %d %d %f %f", ($r-30), ($r-30), $len*$scale, 0, 0, $opts{radius}+($r-30)*cos(3.1416/2-$start*$scale), $opts{radius}-($r-30)*sin(3.1416/2-$start*$scale)));

} else {

push (@paths, sprintf ("M%f %f", $opts{radius}+$r*cos(3.1416/2-$start*$scale), $opts{radius}-$r*sin(3.1416/2-$start*$scale)));

push (@paths, sprintf ("A%f %f %f %d %d %f %f", $r, $r, $len*$scale, 1, 1, $opts{radius}+$r*cos(3.1416/2-$end*$scale), $opts{radius}-$r*sin(3.1416/2-$end*$scale)));

push (@paths, sprintf ("L%f %f", $opts{radius}+($r-30)*cos(3.1416/2-$end*$scale), $opts{radius}-($r-30)*sin(3.1416/2-$end*$scale)));

push (@paths, sprintf ("A%f %f %f %d %d %f %f", ($r-30), ($r-30), $len*$scale, 1, 0, $opts{radius}+($r-30)*cos(3.1416/2-$start*$scale), $opts{radius}-($r-30)*sin(3.1416/2-$start*$scale)));

}

push (@paths, 'Z');

$color = "white" if ($color =~ /^\s*$/);

$svg .= path(\@paths, 'fill-opacity' => 1, stroke => $color, fill => $color);

}

close INF;

}

$r += 50;

## 绘制标尺

$svg .= &circle($opts{radius}, $opts{radius}, $r, stroke => 'black', fill => 'none');

for (my $i = 0; $i < $size; $i += 10000) {

#$svg->circle(cx => $opts{radius}+$gc_list[$i]*cos(3.1416/2-$local_list[$i]), cy => $opts{radius}-$gc_list[$i]*sin(3.1416/2-$local_list[$i]), r => 1);

if ($i%50000 == 0) {

$svg .= line($opts{radius}+$r*cos(3.1416/2-$i*$scale), $opts{radius}-$r*sin(3.1416/2-$i*$scale), $opts{radius}+($r-10)*cos(3.1416/2-$i*$scale), $opts{radius}-($r-10)*sin(3.1416/2-$i*$scale), stroke => 'black', fill => 'black');

$svg .= rotateText($opts{radius}+($r-25)*cos(3.1416/2-$i*$scale), $opts{radius}-($r-25)*sin(3.1416/2-$i*$scale), 360*$i/$size, sprintf("%dKb", $i/1000), stroke =>'black', fill=>'black', 'font-size' => 16);

} else {

$svg .= line($opts{radius}+$r*cos(3.1416/2-$i*$scale), $opts{radius}-$r*sin(3.1416/2-$i*$scale), $opts{radius}+($r-5)*cos(3.1416/2-$i*$scale), $opts{radius}-($r-5)*sin(3.1416/2-$i*$scale), stroke => 'black', fill => 'black');

}

}

$r += 50;

$svg .= &circle($opts{radius}, $opts{radius}, $r, stroke => 'black', fill => 'none');

open (INF, $list_file) or die $!;

while (<INF>) {

chomp;

next if (/^\s*$/ or /^\s*#/);

my ($gene_id, $seq_id, $start, $end, $strand, $len, $feature, $db_ref, $fun, $class, $name, $product) = split (/\t/, $_);

#my ($gene_id, $seq_id, $start, $end, $strand, $len, $db_ref, $feature, $color, $fun, $name, $product) = split (/\t/, $_);

my $color = 'black';

$color = $FUN{$fun} if (exists $FUN{$fun});

if ($feature =~ /ORF/i or $feature =~ /CDS/i or $feature =~ /gene/i or

$feature =~ /pseudo/i or $feature =~ /misc_feature/i) {

if ($feature =~ /pseudo/i or $feature =~ /misc_feature/i) {

$name = "∆" . $name;

}

my @paths;

if ($strand eq '+') {

my $rr = $r + 22;

push (@paths, sprintf ("M%f %f", $opts{radius}+$rr*cos(3.1416/2-$start*$scale), $opts{radius}-$rr*sin(3.1416/2-$start*$scale)));

push (@paths, sprintf ("A%f %f %f %d %d %f %f", $rr, $rr, ($len-$opts{mingene})*$scale, 0, 1, $opts{radius}+$rr*cos(3.1416/2-($end-$opts{mingene})*$scale), $opts{radius}-$rr*sin(3.1416/2-($end-$opts{mingene})*$scale))) if ($len > $opts{mingene});

push (@paths, sprintf ("L%f %f", $opts{radius}+($rr-10)*cos(3.1416/2-$end*$scale), $opts{radius}-($rr-10)*sin(3.1416/2-$end*$scale)));

if ($len > $opts{mingene}) {

push (@paths, sprintf ("L%f %f", $opts{radius}+($rr-20)*cos(3.1416/2-($end-$opts{mingene})*$scale), $opts{radius}-($rr-20)*sin(3.1416/2-($end-$opts{mingene})*$scale)));

#push (@paths, sprintf ("A%f %f %f %d %d %f %f", ($r-30), ($r-30), ($len-$opts{mingene})*$scale, 0, 0, $opts{radius}+($r-30)*cos(3.1416/2-$start*$scale), $opts{radius}-($r-30)*sin(3.1416/2-$start*$scale))) if ($len > $opts{mingene});

push (@paths, sprintf ("A%f %f %f %d %d %f %f", ($rr-20), ($r-20), ($len-$opts{mingene})*$scale, 0, 0, $opts{radius}+($rr-20)*cos(3.1416/2-$start*$scale), $opts{radius}-($rr-20)*sin(3.1416/2-$start*$scale))) if ($len > $opts{mingene});

} else {

push (@paths, sprintf ("L%f %f", $opts{radius}+($rr-20)*cos(3.1416/2-$start*$scale), $opts{radius}-($rr-20)*sin(3.1416/2-$start*$scale)));

}

push (@paths, 'Z');

#$svg .= path(\@paths, 'fill-opacity' => 100, stroke => $color, fill => $color);

} else {

push (@paths, sprintf ("M%f %f", $opts{radius}+$r*cos(3.1416/2-$end*$scale), $opts{radius}-$r*sin(3.1416/2-$end*$scale)));

push (@paths, sprintf ("A%f %f %f %d %d %f %f", $r, $r, ($len-$opts{mingene})*$scale, 0, 0, $opts{radius}+$r*cos(3.1416/2-($start+$opts{mingene})*$scale), $opts{radius}-$r*sin(3.1416/2-($start+$opts{mingene})*$scale))) if ($len > $opts{mingene});

push (@paths, sprintf ("L%f %f", $opts{radius}+($r-10)*cos(3.1416/2-$start*$scale), $opts{radius}-($r-10)*sin(3.1416/2-$start*$scale)));

push (@paths, sprintf ("L%f %f", $opts{radius}+($r-20)*cos(3.1416/2-($start+$opts{mingene})*$scale), $opts{radius}-($r-20)*sin(3.1416/2-($start+$opts{mingene})*$scale))) if ($len > $opts{mingene});

push (@paths, sprintf ("A%f %f %f %d %d %f %f", ($r-20), ($r-20), ($len-$opts{mingene})*$scale, 0, 1, $opts{radius}+($r-20)*cos(3.1416/2-$end*$scale), $opts{radius}-($r-20)*sin(3.1416/2-$end*$scale))) if ($len > $opts{mingene});

push (@paths, sprintf ("L%f %f", $opts{radius}+($r-20)*cos(3.1416/2-$end*$scale), $opts{radius}-($r-20)*sin(3.1416/2-$end*$scale)));

push (@paths, 'Z');

}

$color = "white" if ($color =~ /^\s*$/);

$svg .= path(\@paths, 'fill-opacity' => 1, stroke => 'black', fill => $color);

if (cos(3.1416/2-($start+$end)/2*$scale) > 0) {

$svg .= rotateText($opts{radius}+($r+40)*cos(3.1416/2-($start+$end)/2*$scale), $opts{radius}-($r+40)*sin(3.1416/2-($start+$end)/2*$scale), 360*($start+$end)/2/$size-90, $name, stroke =>'black', fill=>'black', 'font-size' => 18, 'font-style'=>'italic') if (defined $name and !exists $opts{unname});

} else {

$svg .= rotateText($opts{radius}+($r+70)*cos(3.1416/2-($start+$end)/2*$scale), $opts{radius}-($r+70)*sin(3.1416/2-($start+$end)/2*$scale), 360*($start+$end)/2/$size-270, $name, stroke =>'black', fill=>'black', 'font-size' => 18, 'font-style'=>'italic') if (defined $name and !exists $opts{unname});

}

}

}

close INF;

if (exists $opts{desc}) {

$r += 50;

open (IN, $opts{desc}) or die "failed to open file $opts{desc}\n";

while (<IN>) {

chomp;

next if (/^\s*$/ or /^\s*#/);

my ($seq_id, $start, $end, $strand, $len, $desc, $color) = split (/\t/, $_, 7);

my @paths;

push (@paths, sprintf ("M%f %f", $opts{radius}+($r-10)*cos(3.1416/2-$start*$scale), $opts{radius}-($r-10)*sin(3.1416/2-$start*$scale)));

push (@paths, sprintf ("L%f %f", $opts{radius}+$r*cos(3.1416/2-$start*$scale), $opts{radius}-$r*sin(3.1416/2-$start*$scale)));

push (@paths, sprintf ("A%f %f %f %d %d %f %f", $r, $r, $len*$scale, 0, 1, $opts{radius}+$r*cos(3.1416/2-$end*$scale), $opts{radius}-$r*sin(3.1416/2-$end*$scale)));

push (@paths, sprintf ("L%f %f", $opts{radius}+($r-10)*cos(3.1416/2-$end*$scale), $opts{radius}-($r-10)*sin(3.1416/2-$end*$scale)));

push (@paths, sprintf ("A%f %f %f %d %d %f %f", $r-10, $r-10, $len*$scale, 0, 0, $opts{radius}+($r-10)*cos(3.1416/2-$start*$scale), $opts{radius}-($r-10)*sin(3.1416/2-$start*$scale)));

push (@paths, 'Z');

$svg .= path(\@paths, 'stroke-width' => 2, 'fill-opacity' => 1, stroke => 'black', fill => $color);

=cut

my $mean = ($start+$end)/2;

$svg .= rotateText($opts{radius}+($r+20)*cos(3.1416/2-$start*$scale), $opts{radius}-($r+20)*sin(3.1416/2-$start*$scale), 360*$mean/$size, $desc, stroke =>'black', fill=>'black', 'font-size' => 18);

my @path = ();

push (@path, sprintf ("M%f %f", $opts{radius}+($r+20)*cos(3.1416/2-$start*$scale), $opts{radius}-($r+20)*sin(3.1416/2-$start*$scale)));

push (@path, sprintf ("A%f %f %f %d %d %f %f", $r+20, $r+20, $len*$scale, 0, 1, $opts{radius}+($r+20)*cos(3.1416/2-$end*$scale), $opts{radius}-($r+20)*sin(3.1416/2-$end*$scale)));

$svg .= &defPath($start, \@path);

$svg .= &pathText($opts{radius}+($r+20)*cos(3.1416/2-$start*$scale), $opts{radius}-$r*sin(3.1416/2-$start*$scale), $start, $desc, stroke =>$color, fill=>$color, 'font-size' => 18, 'font-style'=>'italic');

=cut

if (cos(3.1416/2-($start+$end)/2*$scale) > 0) {

$svg .= rotateText($opts{radius}+($r+20)*cos(3.1416/2-($start+$end)/2*$scale), $opts{radius}-($r+20)*sin(3.1416/2-($start+$end)/2*$scale), 360*($start+$end)/2/$size-90, $desc, stroke =>'black', fill=>'black', 'font-size' => 18, 'font-style'=>'italic') if (defined $desc);

} else {

$svg .= rotateText($opts{radius}+($r+50)*cos(3.1416/2-($start+$end)/2*$scale), $opts{radius}-($r+50)*sin(3.1416/2-($start+$end)/2*$scale), 360*($start+$end)/2/$size-270, $desc, stroke =>'black', fill=>'black', 'font-size' => 18, 'font-style'=>'italic') if (defined $desc);

}

}

close IN;

}

#silvery

my ($xx, $yy);

my ($b_n, $a_n) = (0, 0);

$svg .= text(30, 2*$opts{radius}+50, 'Backbone', stroke => 'black', fill=>'black', 'font-size' => 18);

$svg .= text(430, 2*$opts{radius}+50, 'Accessory modules', stroke => 'black', fill=>'black', 'font-size' => 18);

foreach my $key (sort keys %FUN) {

my $text = $key;

$text =~ s/(\S.+:\s*)//;

if ($key =~ /Backbone/i) {

$xx = 150;

$yy = 2*$opts{radius}+25*$b_n;

$b_n ++;

} else {

$xx = 600;

$yy = 2*$opts{radius}+25*$a_n;

$a_n ++;

}

$svg .= rect($xx, $yy, 15, 20, stroke => 'black',

fill => $FUN{$key}); #my ($x, $y, $width, $height, %style) = @_;

$svg .= text($xx+30, $yy + 15, $text, stroke => 'black', fill=>'black', 'font-size' => 18);

}

my @paths;

push (@paths, sprintf ("M%f %f", 140, 2*$opts{radius}));

push (@paths, sprintf ("L%f %f", 130, 2*$opts{radius}));

push (@paths, sprintf ("L%f %f", 130, 2*$opts{radius}+25*$b_n-5));

push (@paths, sprintf ("L%f %f", 140, 2*$opts{radius}+25*$b_n-5));

$svg .= path(\@paths, 'stroke-width' => 2, 'fill-opacity' => 1, stroke => 'black', fill => 'none');

@paths = ();

push (@paths, sprintf ("M%f %f", 590, 2*$opts{radius}));

push (@paths, sprintf ("L%f %f", 580, 2*$opts{radius}));

push (@paths, sprintf ("L%f %f", 580, 2*$opts{radius}+25*$a_n-5));

push (@paths, sprintf ("L%f %f", 590, 2*$opts{radius}+25*$a_n-5));

$svg .= path(\@paths, 'stroke-width' => 2, 'fill-opacity' => 1, stroke => 'black', fill => 'none');

#$svg .= circle($opts{radius}, $opts{radius}, 150, stroke=>'gray', fill=>'none');

#$svg .= circle($opts{radius}, $opts{radius}, 200, stroke=>'gray', fill=>'none');

#$svg .= circle($opts{radius}, $opts{radius}, 280, stroke=>'gray', fill=>'none');

$svg .= &end();

open (STDOUT, ">$opts{output}") or die $! if (exists $opts{output});

print "$svg";

close STDOUT;

sub gc_skew {

my $str = shift;

my $len = length $str;

my $num_a = ($str =~ s/([Aa])/$1/g);

my $num_t = ($str =~ s/([Tt])/$1/g);

my $num_c = ($str =~ s/([Cc])/$1/g);

my $num_g = ($str =~ s/([Gg])/$1/g);

my $gc = ($num_g+$num_c)/($num_a+$num_t+$num_c+$num_g);

my $skew = ($num_g-$num_c)/($num_g+$num_c);

return ($gc, $skew);

}

#style="stroke:#323232;stroke-opacity:1;stroke-width:1;stroke-linejoin:miter;stroke-miterlimit:2;stroke-linecap:round;fill-rule:evenodd;fill:#749624;fill-opacity:0.784314;"

sub usage {

my $flag = shift;

print qq(@_

PROGRAM

$Script - $VERSION

USAGE

perl $0 [options] <sequence.fasta> <geneTable.list>

ARGUMENTS

<sequence.fasta> input genome sequence file by fasta format

<geneTable.list> input gene table file by list format

OPTIONS

-output <STR> output file to instead of [STDOUT]

-color <STR> function category color list [$opts{color}]

-frame <STR> frame struct list [none]

-desc <STR> description infomation [none]

-title <STR> title string [$opts{title}]

-window <INT> windows width [$opts{window}]

-radius <INT> circular radius [$opts{radius}]

-step <INT> step length [$opts{step}]

-mingene<INT> min gene length [$opts{mingene}]

-unname donot display gene name

DESCRIPTION

This script using for plot plasmid circular figure.

<geneTable.list> format:

[01] gene_id

[02] seq_id

[03] start

[04] end

[05] strand

[06] length

[07] db_xref

[08] frame_struct

[09] function_category

[10] gene_name

[11] product

\n);

exit;

}

sub new {

my ($width, $height) = @_;

$width = '100%' if (!defined $width);

$height = '100%' if (!defined $height);

return sprintf ("<?xml version=\"1.0\" standalone=\"no\"?>

<!DOCTYPE svg PUBLIC \"-//W3C//DTD SVG 1.1//EN\" \"http://www.w3.org/Graphics/SVG/1.1/DTD/svg11.dtd\">

<svg width=\"$width\" height=\"$height\" version=\"1.1\" xmlns=\"http://www.w3.org/2000/svg\"

xmlns:xlink=\"http://www.w3.org/1999/xlink\">\n");

}

sub ellipse {

my ($cx, $cy, $rx, $ry, %style) = @_;

return sprintf ("<ellipse cx=\"$cx\" cy=\"$cy\" rx=\"$rx\" ry=\"$ry\" style=\"%s\" />\n", join(";", map("$_:$style{$_}", keys %style)));

}

sub circle {

my ($cx, $cy, $r, %style) = @_;

return sprintf ("<circle cx=\"$cx\" cy=\"$cy\" r=\"$r\" style=\"%s\" />\n", join(";", map("$_:$style{$_}", keys %style)));

}

sub line {

my ($x1, $y1, $x2, $y2, %style) = @_;

return sprintf ("<line x1=\"$x1\" y1=\"$y1\" x2=\"$x2\" y2=\"$y2\" style=\"%s\" />\n", join(";", map("$_:$style{$_}", keys %style)));

}

sub rect {

my ($x, $y, $width, $height, %style) = @_;

return sprintf ("<rect x=\"$x\" y=\"$y\" width=\"$width\" height=\"$height\" style=\"%s\" />\n", join(";", map("$_:$style{$_}", keys %style)));

}

sub polygon {

my ($points, %style) = @_;

return sprintf ("<polygon points=\"%s\" style=\"%s\" />\n", join(" ", @$points), join(";", map("$_:$style{$_}", keys %style)));

}

sub polyline {

my ($points, %style) = @_;

return sprintf ("<polyline points=\"%s\" style=\"%s\" />\n", join(" ", @$points), join(";", map("$_:$style{$_}", keys %style)));

}

sub path {

my ($paths, %style) = @_;

return sprintf ("<path d=\"%s\" style=\"%s\"/> \n", join("\n", @$paths), join(";", map("$_:$style{$_}", keys %style)));

}

sub defPath {

my ($id, $paths) = @_;

return sprintf ("<defs> <path id=\"p$id\" d=\"%s\"/> </defs>\n", join("\n", @$paths));

}

sub text {

my ($x, $y, $text, %style) = @_;

return sprintf ("<text x=\"$x\" y=\"$y\" style=\"%s\">$text</text>\n", join(";", map("$_:$style{$_}", keys %style)));

}

sub rotateText {

my ($x, $y, $angle, $text, %style) = @_;

return sprintf ("<text x=\"$x\" y=\"$y\" transform=\"rotate($angle $x,$y)\" style=\"%s\">$text</text>\n", join(";", map("$_:$style{$_}", keys %style)));

}

sub pathText {

my ($x, $y, $path, $text, %style) = @_;

return sprintf ("<text x=\"$x\" y=\"$y\" style=\"%s\"> <textPath xlink:href=\"#p$path\">$text</textPath></text>\n", join(";", map("$_:$style{$_}", keys %style)));

}

sub end {

return sprintf qq(</svg>\n);

}
