# Supplementary material for: Characterization and Comparative Genomics Analysis of lncFII Multi-Resistance Plasmids Carrying blaCTX–M and Type1 Integrons From Escherichia coli
Source: Front Microbiol. 2021 Nov 16;12:753979. doi: 10.3389/fmicb.2021.753979 (PMC8637017; doi:10.3389/fmicb.2021.753979)
Supplement: Supplementary file 1 [file Data_Sheet_1.zip › Supplementary File 2.DOCX]

#!/usr/bin/perl -w

#========================================================================

# Author: Lizhi.Xu

# Email: xulzh@hotmail.com

#========================================================================

use strict;

use warnings;

use FindBin qw($Bin $Script);

#use lib "$FindBin::Bin/lib";

use Getopt::Long;

my $VERSION = "0.2.0";

my %opts = (gene_height => 30, align_height => 150, ratio =>

0.05, border => 20, scale_step => 500, model => 2, length => 500, color =>

'color.cfg');

GetOptions(\%opts, 'output=s', 'color=s', 'model=i', 'legend', 'gene_height=i',

'align_height=i', 'ratio=f', 'scale_step=i', 'length=i',

'border=i', 'scale_plot', 'help') or &usage(2, $!);

# check options

&usage(2, "Show the help message\n") if (exists $opts{help});

&usage(1, "Input was not accepted, use '-help' to get more message\n") if (@ARGV < 1);

my %align_color = ('+' => 'teal', '-' => 'salmon');

my %feature_color;

open (CL, $opts{color}) or die "failed to open: $opts{color}\n";

while (<CL>) {

chomp;

next if (/^\s*$/ or /^\s*#/);

my ($key, $value) = split (/\t/, $_);

$feature_color{$key} = $value;

warn "# $key: $value\n";

}

close CL;

# get config information

my %hash;

my $rank = 0;

foreach my $cfg_file (@ARGV) {

open (CFG, $cfg_file) or die "failed to open: $cfg_file, $!\n";

while (<CFG>) {

chomp;

next if (/^\s*$/ or /^\s*#/);

if (/\[REF\]/) {

$rank ++;

} elsif (/^\s*(\S+)\s*=\s*(\S*)/) {

$hash{$rank}{$1} = $2;

} else {

die "undefined config line: $_\n";

}

}

close CFG;

}

# add sequence size

$/ = "\n>";

foreach my $num (keys %hash) {

$hash{$num}{move} = 0 if (!exists $hash{$num}{move});

$hash{$num}{strand} = '+' if (!exists $hash{$num}{strand});

if (exists $hash{$num}{locus}) {

$hash{$num}{size} = subseqfile("$hash{$num}{name}_locus.fa", $hash{$num}{seq}, $hash{$num}{locus}, $hash{$num}{strand});

$hash{$num}{seq} = "$hash{$num}{name}_locus.fa";

} else {

$hash{$num}{size} = seqLen($hash{$num}{seq});

}

}

$/ = "\n";

my $svg;

my ($x, $y) = (0, $opts{border});

foreach my $num (sort {$a <=> $b} keys %hash) {

#warn "my \$size = subseqfile($hash{$num}{name}_locus.fa, $hash{$num}{seq}, $hash{$num}{locus}, $hash{$num}{strand})\n";

my $size = $hash{$num}{size};

my ($plot_start, $plot_end) = split (/-/, $hash{$num}{locus});

$x = $size if ($x < $size);

if ($num == 1) {

if (exists $opts{scale_plot}) {

$y += 15;

$svg .= scaleIcon($opts{border}+$hash{$num}{move}*$opts{ratio}, $y, $plot_start, $size, $opts{scale_step}, $opts{ratio}, -10, 1);

$y+= 25;

}

# plot chr line

#$svg .= rect($opts{border}, $y, $size*$opts{ratio}, $opts{gene_height}+$opts{space_height}*2, 'stroke-width' => 1, stroke =>'black', fill=>'none');

$hash{$num}{move} = 0 if (!exists $hash{$num}{move});

$svg .= rect($opts{border}+$hash{$num}{move}*$opts{ratio}, $y+$opts{gene_height}/2, $size*$opts{ratio}, 1, 'stroke-width' => 1, stroke =>'black', fill=>'none');

$svg .= text($opts{border}+($hash{$num}{move}+$size)*$opts{ratio}+10, $y+$opts{gene_height}/2, sprintf("$hash{$num}{name} \(%.2fKb\)", $size/1000), stroke =>'black', fill=>'black', 'font-size' => 18);

$y += $opts{gene_height};

} else {

#$y += 5;

#$svg .= line($opts{border}, $y, $opts{border}+$size*$opts{ratio}, $y, 'stroke-width' => 2, stroke =>'black', fill=>'black');

$y += $opts{align_height};

system ("/blast/makeblastdb -in $hash{$num}{seq} -dbtype nucl");

system ("/blast/blastn -db $hash{$num}{seq} -query $hash{$num-1}{seq} -out $hash{$num}{name}_$hash{$num-1}{name}.locus.blast -outfmt 6 -evalue 1e-5");

open (BLAST, "$hash{$num}{name}_$hash{$num-1}{name}.locus.blast") or die

"failed to open file: $hash{$num}{name}_$hash{$num-1}{name}.locus.blast";

while (<BLAST>) {

chomp;

my ($query_id, $subj_id, $identity, $align, $mismatch, $gap, $query_start, $query_end, $subj_start, $subj_end, $evalue, $score) = split (/\t/, $_);

next if ($align < $opts{length});

($query_start, $query_end) = ($query_start+$hash{$num-1}{move}, $query_end+$hash{$num-1}{move});

($subj_start, $subj_end) = ($subj_start+$hash{$num}{move}, $subj_end+$hash{$num}{move});

my $strand = ($query_start<$query_end)?'+':'-';

$strand = ($subj_start<$subj_end)?'+':'-';

#warn "$query_start, $query_end, $subj_start, $subj_end,$strand\n";

my @points = ();

push (@points, sprintf("%f,%f",

$opts{border}+$query_start*$opts{ratio},

$y-$opts{align_height}-$opts{gene_height}/2));

push (@points, sprintf("%f,%f",

$opts{border}+$subj_start*$opts{ratio},

$y+$opts{gene_height}/2));

push (@points, sprintf("%f,%f",

$opts{border}+$subj_end*$opts{ratio},

$y+$opts{gene_height}/2));

push (@points, sprintf("%f,%f",

$opts{border}+$query_end*$opts{ratio},

$y-$opts{align_height}-$opts{gene_height}/2));

$svg .= polygon(\@points, 'fill-opacity' => 0.2, stroke => 'null', fill => $align_color{$strand});

}

close BLAST;

#$svg .= rect($opts{border}, $y, $size*$opts{ratio}, $opts{gene_height}+$opts{space_height}*2, 'stroke-width' => 1, stroke =>'black', fill=>'none');

$svg .= rect($opts{border}+$hash{$num}{move}*$opts{ratio}, $y+$opts{gene_height}/2, $size*$opts{ratio}, 1, 'stroke-width' => 1, stroke =>'black', fill=>'none');

$svg .= text($opts{border}+($hash{$num}{move}+$size)*$opts{ratio}+10, $y+$opts{gene_height}/2, sprintf("$hash{$num}{name} \(%.2fKb\)", $size/1000), stroke =>'black', fill=>'black', 'font-size' => 18);

$y += $opts{gene_height};

#$svg .= line($opts{border}, $y+3, $opts{border}+$size*$opts{ratio}, $y+3, 'stroke-width' => 3, stroke =>'black', fill=>'black');

}

}

($x, $y) = (0, $opts{border});

foreach my $num (sort {$a <=> $b} keys %hash) {

my $size = $hash{$num}{size};

my ($plot_start, $plot_end) = split (/-/, $hash{$num}{locus});

$x = $size if ($x < $size);

if ($num == 1) {

if (exists $opts{scale_plot}) {

$y += 25;

#$svg .= scaleIcon($opts{border}, $y, $size, $opts{step}, $opts{ratio}, -10, 1);

$y+= 15;

}

# plot chr line

#$svg .= rect($opts{border}, $y, $size*$opts{ratio}, $opts{gene_height}+$opts{space_height}*2, 'stroke-width' => 1, stroke =>'black', fill=>'none');

$y += $opts{gene_height};

open (LST, $hash{$num}{list}) or die "failed to open file: $hash{$num}{list}, $!\n";

while (<LST>) {

chomp;

next if (/^\s*$/ or /^\s*#/);

;

#my ($seq_id, $start, $end, $strand, $locus_tag, $len, $db_xref, $feature, $fun, $color, $type, $gene_name, $product) = split (/\t/, $_);

#my ($locus_tag, $seq_id, $start, $end, $strand, $len, $db_xref, $feature, $color, $fun, $gene_name, $product) = split (/\t/, $_);

#my ($seq_id, $start, $end, $strand, $gene_id, $len, $feature, $db_xref, $fun, $color, $gene_name, $product) = split (/\t/, $_);

my ($gene_id, $seq_id, $start, $end, $strand, $len, $feature, $desc, $db_xref, $classify, $gene_name, $product) = split (/\t/, $_);

$start =~ s/\D//g;

$end =~ s/\D//g;

next if ($start < $plot_start or $end > $plot_end);

if ($hash{$num}{strand} eq '-') {

($start, $end) = ($plot_end-$end, $plot_end-$start);

$strand = ($strand eq '+')?'-':'+';

} else {

$start = $start - $plot_start;

$end = $end - $plot_start;

}

if (exists $hash{$num}{move}) {

($start, $end) = ($start+$hash{$num}{move}, $end+$hash{$num}{move});

}

my $mean = ($start + $end)/2;

$classify = "Others" if (length $classify == 0);

# plot gene struct

if ($feature =~ /gene/i or $feature =~ /CDS/i or $feature =~ /ORF/i or $feature =~ /pseudo/i or $feature =~ /misc_feature/i) {

$svg .= geneIcon($opts{border}+$start*$opts{ratio},

$y-$opts{gene_height}, $strand, $len*$opts{ratio},

$opts{gene_height}, $opts{model}, 'fill-opacity' => 0.8,

stroke => 'black', fill => $feature_color{$classify});

#warn "geneIcon($opts{border}+$start*$opts{ratio}, $y-$opts{gene_height}, $strand, $len*$opts{ratio}, $opts{gene_height}, $opts{model}, 'fill-opacity' => 0.8, stroke => 'black', fill => $color";

if (defined $gene_name and $gene_name ne '-') {

if ($feature =~ /pseudo/i or $feature =~ /misc_feature/i) {

$gene_name = '\'' . $gene_name;

}

$svg .= rotateText($opts{border}+$mean*$opts{ratio}-20,

$y+10, 45, $gene_name, 'font-style'=>'italic',

stroke => 'black', fill=> 'black', 'font-size' => 16);

}

} elsif ($feature =~ /mobile_element/ and $product =~ /MITE/i) {

$svg .= rect($opts{border}+$start*$opts{ratio},

$y-$opts{gene_height}, $len*$opts{ratio},

$opts{gene_height}, 'fill-opacity' => 0.8, stroke =>

'black', fill => $feature_color{$classify});

} elsif ($feature =~ /repeat_region/ and $product =~ /direct/i) {

my $dir = '-';

if (($strand =~ /\+/ and $gene_name =~ /DRL/) or ($strand =~ /-/ and $gene_name =~ /DRR/)) {

$dir = '+';

}

$svg .= drIcon($opts{border}+$mean*$opts{ratio},

$y-$opts{gene_height}/2, $dir, $opts{gene_height}/2, 10, 'fill-opacity' =>

1, 'stroke-width' => 1, stroke => $feature_color{$classify}, fill=>

$feature_color{$classify});

} elsif ($feature =~ /repeat_region/ and $product =~ /inverted/i) {

my $dir = '-';

if (($strand =~ /\+/ and $gene_name =~ /IR[Li]/) or ($strand =~ /-/ and $gene_name =~ /IR[Rt]/)) {

$dir = '+';

}

$svg .= irIcon($opts{border}+$mean*$opts{ratio},

$y-$opts{gene_height}/2, $dir, $opts{gene_height}/2, 10,

'fill-opacity' => 1,'stroke-width' => 1, stroke =>

$feature_color{$classify},

fill=> $feature_color{$classify});

} elsif ($feature =~ /regulatory/ and $product =~ /promoter/i) {

$svg .= tranIcon($opts{border}+$mean*$opts{ratio}, $y-$opts{gene_height}/2, $strand, $opts{gene_height}, $opts{gene_height}/2, 'fill-opacity' => 1,'stroke-width' => 1, stroke =>$feature_color{$classify},fill=> 'none');

} elsif ($feature =~ /misc_recomb/ and $product =~ /res/i) {

$svg .= resIcon($opts{border}+$mean*$opts{ratio}, $y-$opts{gene_height}/2, $opts{gene_height}/2, 10, 'fill-opacity' => 1,'stroke-width' => 1, stroke => $feature_color{$classify}, fill=> $feature_color{$classify});

} elsif ($feature =~ /misc_recomb/ and $gene_name =~ /att/i) {

$svg .= attIcon($opts{border}+$mean*$opts{ratio}, $y-$opts{gene_height}/2, $opts{gene_height}/2, 10, 'fill-opacity' => 1,'stroke-width' => 1, stroke =>$feature_color{$classify}, fill=> $feature_color{$classify});

$svg .= rotateText($opts{border}+$mean*$opts{ratio}-20,

$y+10, 45, $gene_name, 'font-style'=>'italic',

stroke => 'black', fill=> 'black', 'font-size' => 16) if (defined $gene_name and $gene_name ne '-');

}

}

close LST;

} else {

$y += $opts{align_height};

$y += $opts{gene_height};

open (LST, $hash{$num}{list}) or die "failed to open file: $hash{$num}{list}\n";

while (<LST>) {

chomp;

next if (/^\s*$/ or /^\s*#/);

my ($gene_id, $seq_id, $start, $end, $strand, $len, $feature, $desc, $db_xref, $classify, $gene_name, $product) = split (/\t/, $_);

$start =~ s/\D//g;

$end =~ s/\D//g;

next if ($start < $plot_start or $end > $plot_end);

if ($hash{$num}{strand} eq '-') {

($start, $end) = ($plot_end-$end, $plot_end-$start);

$strand = ($strand eq '+')?'-':'+';

} else {

$start = $start - $plot_start;

$end = $end - $plot_start;

}

if (exists $hash{$num}{move}) {

($start, $end) = ($start+$hash{$num}{move}, $end+$hash{$num}{move});

}

my $mean = ($start+$end)/2;

$classify = "Others" if (length $classify == 0);

# plot gene struct

if ($feature =~ /gene/i or $feature =~ /CDS/i or $feature =~ /ORF/i or $feature =~ /pseudo/i or $feature =~ /misc_feature/i) {

$svg .= geneIcon($opts{border}+$start*$opts{ratio},

$y-$opts{gene_height}, $strand, $len*$opts{ratio},

$opts{gene_height}, $opts{model}, 'fill-opacity' => 0.8, stroke

=> 'black', fill => $feature_color{$classify});

if (defined $gene_name and $gene_name ne '-') {

if ($feature =~ /pseudo/i) {

$gene_name = '\'' . $gene_name;

}

$svg .= rotateText($opts{border}+$mean*$opts{ratio}-20,

$y+10, 45, $gene_name, 'font-style'=>'italic',

stroke => 'black', fill=> 'black', 'font-size' => 16);

}

} elsif ($feature =~ /mobile_element/ and $product =~ /MITE/i) {

$svg .= rect($opts{border}+$start*$opts{ratio},

$y-$opts{gene_height}*3/4, $len*$opts{ratio},

$opts{gene_height}/2, 'fill-opacity' => 0.8, stroke =>

'black', fill => $feature_color{$classify});

} elsif ($feature =~ /repeat_region/ and $product =~ /direct/i) {

my $dir = '-';

if (($strand eq '+' and $gene_name =~ /DRL/) or ($strand eq '-' and $gene_name =~ /DRR/)) {

$dir = '+';

}

$svg .= drIcon($opts{border}+$mean*$opts{ratio},

$y-$opts{gene_height}/2, $dir, $opts{gene_height}/2, 10, 'fill-opacity' =>

1, 'stroke-width' => 1, stroke => $feature_color{$classify}, fill=>

$feature_color{$classify});

} elsif ($feature =~ /repeat_region/ and $product =~ /inverted/i) {

my $dir = '-';

if (($strand eq '+' and $gene_name =~ /IR[Li]/) or ($strand eq '-' and $gene_name =~ /IR[Rt]/)) {

$dir = '+';

}

$svg .= irIcon($opts{border}+$mean*$opts{ratio},

$y-$opts{gene_height}/2, $dir, $opts{gene_height}/2, 10,

'fill-opacity' => 1,'stroke-width' => 1, stroke =>

$feature_color{$classify},

fill=> $feature_color{$classify});

} elsif ($feature =~ /regulatory/ and $product =~ /promoter/i) {

$svg .= tranIcon($opts{border}+$mean*$opts{ratio}, $y-$opts{gene_height}/2, $strand, $opts{gene_height}, $opts{gene_height}/2, 'fill-opacity' => 1,'stroke-width' => 1, stroke => $feature_color{$classify}, fill=> 'none');

} elsif ($feature =~ /misc_recomb/ and $product =~ /res/i) {

$svg .= resIcon($opts{border}+$mean*$opts{ratio}, $y-$opts{gene_height}/2, $opts{gene_height}/2, 10, 'fill-opacity' => 1,'stroke-width' => 1, stroke => $feature_color{$classify}, fill=> $feature_color{$classify});

} elsif ($feature =~ /misc_recomb/ and $gene_name =~ /att/i) {

$svg .= attIcon($opts{border}+$mean*$opts{ratio}, $y-$opts{gene_height}/2, $opts{gene_height}/2, 10, 'fill-opacity' => 1,'stroke-width' => 1, stroke =>$feature_color{$classify},fill=> $feature_color{$classify});

$svg .= rotateText($opts{border}+$mean*$opts{ratio}-20,

$y+10, 45, $gene_name, 'font-style'=>'italic',

stroke => 'black', fill=> 'black', 'font-size' => 16) if (defined $gene_name and $gene_name ne '-');

}

}

close LST;

}

}

my ($xx, $yy);

my ($b_n, $a_n) = (0, 0);

foreach my $key (sort keys %feature_color) {

my $text = $key;

$text =~ s/^\S.+:\s*//;

if ($key =~ /Backbone/i) {

$xx = 150;

$yy = 100 + $y + ($opts{gene_height}+5)*$b_n;

$b_n ++;

} else {

$xx = 600;

$yy = 100 + $y + ($opts{gene_height}+5)*$a_n;

$a_n ++;

}

$svg .= geneIcon($opts{border}+$xx,

$yy-$opts{gene_height}, '+', 30,

$opts{gene_height}, $opts{model}, 'fill-opacity' => 0.8, stroke

=> 'black', fill => $feature_color{$key});

$svg .= text($opts{border}+$xx+50, $yy-$opts{gene_height}/4,

$text, stroke =>'black', fill=>'black', 'font-size' => 18);

}

$svg .= text($opts{border}+20, $y+($opts{gene_height}+5)*$a_n/2,

'Backbone', stroke =>'black', fill=>'black', 'font-size' => 18);

$svg .= text($opts{border}+400, $y+($opts{gene_height}+5)*$a_n/2,

'Accessory modules', stroke =>'black', fill=>'black', 'font-size' => 18);

my @paths;

push (@paths, sprintf ("M%f %f", 140, $y+70));

push (@paths, sprintf ("L%f %f", 130, $y+70));

push (@paths, sprintf ("L%f %f", 130, $y+70+($opts{gene_height}+5)*$b_n-5));

push (@paths, sprintf ("L%f %f", 140, $y+70+($opts{gene_height}+5)*$b_n-5));

$svg .= path(\@paths, 'stroke-width' => 2, 'fill-opacity' => 1, stroke =>'black', fill => 'none');

@paths = ();

push (@paths, sprintf ("M%f %f", 590, $y+70));

push (@paths, sprintf ("L%f %f", 580, $y+70));

push (@paths, sprintf ("L%f %f", 580, $y+70+($opts{gene_height}+5)*$a_n-5));

push (@paths, sprintf ("L%f %f", 590, $y+70+($opts{gene_height}+5)*$a_n-5));

$svg .= path(\@paths, 'stroke-width' => 2, 'fill-opacity' => 1, stroke =>'black', fill => 'none');

if ($b_n > $a_n) {

$y = 100 + $y + ($opts{gene_height}+5)*$b_n;

} else {

$y = 100 + $y + ($opts{gene_height}+5)*$a_n;

}

open (STDOUT, ">$opts{output}") or die $! if (exists $opts{output});

print writeSVG($x*$opts{ratio}+$opts{border}*2+120, $y+$opts{border}, $svg);

close STDOUT;

sub subseqfile {

my ($outfile, $infile, $locus, $strand) = @_;

my ($start, $end) = split(/-/, $locus);

my $len = $end-$start+1;

open (OUT, ">$outfile") or die "error: failed to open $outfile\n";

open (IN, $infile) or die "error: failed to open $infile\n";

$/ = "\n>";

while (<IN>) {

chomp;

s/^>//g;

next if (/^\s*$/);

my ($head, $seq) = split (/\n+/, $_, 2);

my ($id, $desc) = split (/\s+/, $head, 2);

$seq =~ s/\s+//g;

$seq = substr($seq, $start-1, $len);

$seq =~ s/(\w{60})/$1\n/g;

$seq =~ s/\n+$//;

if ($strand eq '-') {

$seq =~ tr/ATGCN/TACGN/;

$seq = reverse $seq;

}

print OUT ">$id:$locus\n$seq\n";

}

$/ = "\n";

close IN;

close OUT;

return $len;

}

sub seqLen {

my $seq_file = shift;

my $len = 0;

open (SEQ, $seq_file) or die "failed to open: $seq_file\n";

$/ = "\n>";

while (<SEQ>) {

chomp;

s/^>//g;

next if (/^\s*$/);

my ($head, $seq) = split (/\n/, $_, 2);

my ($id, $desc) = split (/\s+/, $head, 2);

$seq =~ s/\s+//g;

$len += length $seq;

}

$/ = "\n";

close SEQ;

return $len;

}

sub usage {

my $flag = shift;

print qq(@_

PROGRAM

$Script - $VERSION

USAGE

perl $0 [options] <input.cfg>

ARGUMENTS

<input> input config set file

OPTIONS

-output <File> output file to instead of [STDOUT]

-color <Str> function category color list [$opts{color}]

-ratio <Float> ratio for width [$opts{ratio}]

-border <Int> border width [$opts{border}]

-model <Int> gene icon model [$opts{model}]

-length <Int> min align length [$opts{length}]

-gene_height <Int> gene icon height [$opts{gene_height}]

-align_height<Int> align height [$opts{align_height}]

-scale_step <Int> scale step set [$opts{scale_step}]

-scale_plot plot scale at head

DESCRIPTION

This script using for plot locus align figure.

<input.cfg>

[REF]

name=ref1

locus=<start>-<end>

strand=[+/-]

move=<size>

seq=ref.fa

list=ref_genes.list

[REF]

...

\n);

exit;

}

sub writeSVG {

my ($width, $height, $svg) = @_;

return sprintf ("<?xml version=\"1.0\" standalone=\"no\"?>

<!DOCTYPE svg PUBLIC \"-//W3C//DTD SVG 1.1//EN\" \"http://www.w3.org/Graphics/SVG/1.1/DTD/svg11.dtd\">

<svg width=\"$width\" height=\"$height\" version=\"1.1\" xmlns=\"http://www.w3.org/2000/svg\">

$svg

</svg>\n");

}

sub scaleIcon {

my ($x, $y, $start, $size, $step, $ratio, $height, $flag) = @_;

my $xml = line($x, $y, $x+$size*$ratio, $y, stroke =>'black', fill=>'black');

for (my $i = 0; $i < $size; $i += $step) {

if ($i%($step*10) == 0) {

$xml .= line($x+$i*$ratio, $y, $x+$i*$opts{ratio}, $y+$height, stroke =>'black', fill=>'black');

$xml .= text($x+$i*$ratio, $y+$height, sprintf ("%dKb", ($start+$i)/1000), stroke =>'black', fill=>'black', 'font-size' => 16) if ($flag);

} else {

$xml .= line($x+$i*$ratio, $y, $x+$i*$ratio, $y+$height/2, stroke =>'black', fill=>'black');

}

}

return $xml;

}

sub geneIcon {

my ($x, $y, $strand, $width, $height, $model, %style) = @_;

my @points = ();

if ($strand eq '+') {

if ($model == 1) {

push (@points, sprintf("%f,%f", $x, $y));

push (@points, sprintf("%f,%f", ($width>$height/3)?$x+$width-$height/3:$x, $y));

push (@points, sprintf("%f,%f", $x+$width, $y+$height/2));

push (@points, sprintf("%f,%f", ($width>$height/3)?$x+$width-$height/3:$x, $y+$height));

push (@points, sprintf("%f,%f", $x, $y+$height));

} elsif ($model == 2) {

push (@points, sprintf("%f,%f", $x, $y+$height/4));

push (@points, sprintf("%f,%f", ($width>$height/3)?$x+$width-$height/3:$x, $y+$height/4));

push (@points, sprintf("%f,%f", ($width>$height/3)?$x+$width-$height/3:$x, $y));

push (@points, sprintf("%f,%f", $x+$width, $y+$height/2));

push (@points, sprintf("%f,%f", ($width>$height/3)?$x+$width-$height/3:$x, $y+$height));

push (@points, sprintf("%f,%f", ($width>$height/3)?$x+$width-$height/3:$x, $y+$height*3/4));

push (@points, sprintf("%f,%f", $x, $y+$height*3/4));

} else {

die "Undifined model option '$model'.\n";

}

} else {

if ($model == 1) {

push (@points, sprintf("%f,%f", $x, $y+$height/2));

push (@points, sprintf("%f,%f", ($width>$height/3)?$x+$height/3:$x+$width, $y));

push (@points, sprintf("%f,%f", $x+$width, $y));

push (@points, sprintf("%f,%f", $x+$width, $y+$height));

push (@points, sprintf("%f,%f", ($width>$height/3)?$x+$height/3:$x+$width, $y+$height));

} elsif ($model == 2) {

push (@points, sprintf("%f,%f", $x, $y+$height/2));

push (@points, sprintf("%f,%f", ($width>$height/3)?$x+$height/3:$x+$width, $y));

push (@points, sprintf("%f,%f", ($width>$height/3)?$x+$height/3:$x+$width, $y+$height/4));

push (@points, sprintf("%f,%f", $x+$width, $y+$height/4));

push (@points, sprintf("%f,%f", $x+$width, $y+$height*3/4));

push (@points, sprintf("%f,%f", ($width>$height/3)?$x+$height/3:$x+$width, $y+$height*3/4));

push (@points, sprintf("%f,%f", ($width>$height/3)?$x+$height/3:$x+$width, $y+$height));

} else {

die "Undifined model option '$model'.\n";

}

}

return polygon(\@points, %style);

}

sub drIcon {

my ($x, $y, $strand, $height, $size, %style) = @_;

my $xml = '';

# $xml .= line($x, $y, $x, $y-$height, %style);

my @points = ();

if ($strand eq '+') {

push (@points, sprintf("%f,%f", $x, $y));

push (@points, sprintf("%f,%f", $x-($size/2+2), $y-$height));

push (@points, sprintf("%f,%f", $x-($size/2+2)+$size/2, $y-$height-$size/2));

push (@points, sprintf("%f,%f", $x-($size/2+2), $y-$height-$size));

push (@points, sprintf("%f,%f", $x-($size/2+2)-$size/2, $y-$height-$size/2));

push (@points, sprintf("%f,%f", $x-($size/2+2), $y-$height));

} else {

push (@points, sprintf("%f,%f", $x, $y));

push (@points, sprintf("%f,%f", $x+($size/2+2), $y-$height));

push (@points, sprintf("%f,%f", $x+($size/2+2)+$size/2, $y-$height-$size/2));

push (@points, sprintf("%f,%f", $x+($size/2+2), $y-$height-$size));

push (@points, sprintf("%f,%f", $x+($size/2+2)-$size/2, $y-$height-$size/2));

push (@points, sprintf("%f,%f", $x+($size/2+2), $y-$height));

}

$xml .= polygon(\@points, %style);

return $xml;

}

sub irIcon {

my ($x, $y, $strand, $height, $size, %style) = @_;

my $xml = '';

# $xml .= line($x, $y, $x, $y-$height, %style);

my @points = ();

if ($strand eq '+') {

push (@points, sprintf("%f,%f", $x, $y));

push (@points, sprintf("%f,%f", $x, $y-$height));

push (@points, sprintf("%f,%f", $x+$size, $y-$height-$size/2));

push (@points, sprintf("%f,%f", $x, $y-$height-$size));

push (@points, sprintf("%f,%f", $x, $y-$height));

} else {

push (@points, sprintf("%f,%f", $x, $y));

push (@points, sprintf("%f,%f", $x, $y-$height));

push (@points, sprintf("%f,%f", $x-$size, $y-$height-$size/2));

push (@points, sprintf("%f,%f", $x, $y-$height-$size));

push (@points, sprintf("%f,%f", $x, $y-$height));

}

$xml .= polygon(\@points, %style);

return $xml;

}

sub attIcon {

my ($x, $y, $height, $size, %style) = @_;

my $xml = '';

$xml .= line($x, $y, $x, $y-$height, %style);

$xml .= circle($x, $y-$height-$size/2, $size/2, %style);

return $xml;

}

sub resIcon {

my ($x, $y, $height, $size, %style) = @_;

my $xml = '';

my @points = ();

push (@points, sprintf("%f,%f", $x, $y));

push (@points, sprintf("%f,%f", $x, $y-$height));

push (@points, sprintf("%f,%f", $x+$size/2, $y-$height));

push (@points, sprintf("%f,%f", $x, $y-$height-$size));

push (@points, sprintf("%f,%f", $x-$size/2, $y-$height));

push (@points, sprintf("%f,%f", $x, $y-$height));

$xml .= polygon(\@points, %style);

return $xml;

}

sub signalIcon {

my ($x, $y, $height, $size, $tag, %style) = @_;

my $xml = '';

$xml .= line($x, $y, $x, $y-$height, %style);

$xml .= rect($x-$size, $y-$height-$size, $size*2, $size, %style);

$xml .= text($x-$size+4, $y-$height-2, $tag, 'font-size' => $size, %style);

return $xml;

}

sub tranIcon {

my ($x, $y, $strand, $height, $size, %style) = @_;

my $xml = '';

if ($strand eq '+') {

my @points = ();

push (@points, sprintf("%f,%f", $x, $y));

push (@points, sprintf("%f,%f", $x, $y-$height));

push (@points, sprintf("%f,%f", $x+$size, $y-$height));

$xml .= polyline (\@points, %style);

@points = ();

push (@points, sprintf("%f,%f", $x+$size, $y-$height));

push (@points, sprintf("%f,%f", $x+$size-4, $y-$height-2));

push (@points, sprintf("%f,%f", $x+$size-4, $y-$height+2));

$xml .= polygon(\@points, %style);

} else {

my @points = ();

push (@points, sprintf("%f,%f", $x, $y));

push (@points, sprintf("%f,%f", $x, $y-$height));

push (@points, sprintf("%f,%f", $x-$size, $y-$height));

$xml .= polyline (\@points, %style);

@points = ();

push (@points, sprintf("%f,%f", $x-$size, $y-$height));

push (@points, sprintf("%f,%f", $x-$size+4, $y-$height-2));

push (@points, sprintf("%f,%f", $x-$size+4, $y-$height+2));

$xml .= polygon(\@points, %style);

}

return $xml;

}

sub ellipse {

my ($cx, $cy, $rx, $ry, %style) = @_;

return sprintf ("<ellipse cx=\"$cx\" cy=\"$cy\" rx=\"$rx\" ry=\"$ry\" style=\"%s\" />\n", join(";", map("$_:$style{$_}", keys %style)));

}

sub circle {

my ($cx, $cy, $r, %style) = @_;

return sprintf ("<circle cx=\"$cx\" cy=\"$cy\" r=\"$r\" style=\"%s\" />\n", join(";", map("$_:$style{$_}", keys %style)));

}

sub line {

my ($x1, $y1, $x2, $y2, %style) = @_;

return sprintf ("<line x1=\"$x1\" y1=\"$y1\" x2=\"$x2\" y2=\"$y2\" style=\"%s\" />\n", join(";", map("$_:$style{$_}", keys %style)));

}

sub rect {

my ($x, $y, $width, $height, %style) = @_;

return sprintf ("<rect x=\"$x\" y=\"$y\" width=\"$width\" height=\"$height\" style=\"%s\" />\n", join(";", map("$_:$style{$_}", keys %style)));

}

sub polygon {

my ($points, %style) = @_;

return sprintf ("<polygon points=\"%s\" style=\"%s\" />\n", join(" ", @$points), join(";", map("$_:$style{$_}", keys %style)));

}

sub polyline {

my ($points, %style) = @_;

return sprintf ("<polyline points=\"%s\" style=\"%s\" />\n", join(" ", @$points), join(";", map("$_:$style{$_}", keys %style)));

}

sub path {

my ($paths, %style) = @_;

return sprintf ("<path d=\"%s\" style=\"%s\"/> \n", join("\n", @$paths), join(";", map("$_:$style{$_}", keys %style)));

}

sub text {

my ($x, $y, $text, %style) = @_;

return sprintf ("<text x=\"$x\" y=\"$y\" style=\"%s\">$text</text>\n", join(";", map("$_:$style{$_}", keys %style)));

}

sub rotateText {

my ($x, $y, $angle, $text, %style) = @_;

return sprintf ("<text x=\"$x\" y=\"$y\" transform=\"rotate($angle $x,$y)\" style=\"%s\">$text</text>\n", join(";", map("$_:$style{$_}", keys %style)));

}

sub endsvg {

return sprintf qq(</svg>\n);

}
