# Supplementary material for: Characterization and Comparative Genomics Analysis of lncFII Multi-Resistance Plasmids Carrying blaCTX–M and Type1 Integrons From Escherichia coli
Source: Front Microbiol. 2021 Nov 16;12:753979. doi: 10.3389/fmicb.2021.753979 (PMC8637017; doi:10.3389/fmicb.2021.753979)
Supplement: Supplementary file 1 [file Data_Sheet_1.zip › Supplementary Tables S1-S6 and Figure S1.docx]

***Supplementary Material***

**Table S1 The number of *E. coli* isolates per farm**

| Source | Farm 1 | Farm 2 | Farm 3 | Farm 4 | Farm 5 | Farm 6 |
| --- | --- | --- | --- | --- | --- | --- |
| Isolates | 35 | 34 | 34 | 34 | 34 | 35 |
| Faeces | 10 | 10 | 10 | 10 | 10 | 10 |
| Urine | 10 | 10 | 10 | 10 | 10 | 10 |
| Soil | 8 | 7 | 8 | 7 | 6 | 8 |
| Water | 7 | 7 | 6 | 7 | 8 | 7 |

**Table S2 Disc diffusion method (K-B method) *Escherichia coli* quality control range**

| Category | Antimicrobial  Agent | Disk Content | Interpretive Categories and Zone Diameter Breakpoints (nearest whole mm) | | |
| --- | --- | --- | --- | --- | --- |
|  |  |  | S | I | R |
| First generation cephalosporins | Cefazolin | 30 μg | ≥28 | 21-27 | ≤20 |
| Third generation cephalosporins | Ceftazidime | 30 μg | ≥33 | 25-32 | ≤24 |
| Carbapenem | Imipenem | 10 μg | ≥33 | 26-32 | ≤25 |

**Table S3 The primers of carbapenemases gene segment**

| Enzyme production | Resistance gene | Primer | Product length(bp) | | Annealing temperature  (℃) | | | References |
| --- | --- | --- | --- | --- | --- | --- | --- | --- |
| A | *bla*_GES_ | GES-F: GCTTCATTCACGCACTATT  GES-R: CGATGCTAGAAACCGCTC | | 323 | | 52 | (Hong et al., 2012) | |
|  | *bla*_KPC_ | KPC-F: GTATCGCCGTCTAGTTCTGC  KPC-R: GGTCGTGTTTCCCTTTAGCC | | 638 | | 56 | (Hong et al., 2012) | |
|  | *bla*_SME_ | SMEF1: GAGGAAGACTTTGATGGGAGGAT  SME-R1: TCCCCTCAGGACCGCCAAG | | 334 | | 52 | (Voets et al., 2011) | |
|  | *bla*_IMI_ | IMI-F: TGCGGTCGATTGGAGATAAA  IMI-R: CGATTCTTGAAGCTTCTGCG | | 399 | | 52 | (Hong et al., 2012) | |
|  | *bla*_BIC_ | BIC-F: TATGCAGCTCCTTTAAGGGC  BIC-R: TCATTGGCGGTGCCGTACAC | | 537 | | 52 | (Poirel et al., 2011) | |
| B | *bla*_IMP_ | IMP-F: GGAATAGAGTGGCTTAAYTCTC  IMP-R: UAAAACAACCACC | | 232 | | 56 | (Poirel et al., 2011) | |
|  | *bla*_VIM_ | VIM-F: GATGGTGTTTGGTCGCATA  VIM-R: CGAATGCGCAGCACCAG | | 390 | | 52 | (Poirel et al., 2011) | |
|  | *bla*_NDM_ | NDM-F: GGTTTGGCGATCTGGTTTTC  NDM-R: CGGAATGGCTCATCACGATC | | 328 | | 56 | (Poirel et al., 2011) | |
|  | *bla*_SPM_ | SPM-F: AAAATCTGGGTACGCAAACG  SPM-R: ACATTATCCGCTGGAACAGG | | 271 | | 52 | (Poirel et al., 2011) | |
|  | *bla*_DIM_ | DIM-F: GCTTGTCTTCGCTTGCTAACG  DIM-R: CGTTCGGCTGGATTGATTTG | | 699 | | 52 | (Poirel et al., 2011) | |
|  | *bla*_GIM_ | GIM-F: TCGACACACCTTGGTCTGAA  GIM-R: AACTTCCAACTTTGCCATGC | | 477 | | 52 | (Poirel et al., 2011) | |
|  | *bla*_SIM_ | SIM-F: TACAAGGGATTCGGCATCG  SIM-R: TAATGGCCTGTTCCCATGTG | | 570 | | 52 | (Poirel et al., 2011) | |
| D | *bla*_OXA-23_ | OXA-23-F: GATCGGATTGGAGAACCAGA  OXA-23-R: ATTTCTGACCGCATTTCCAT | | 501 | | 56 | (Woodford et al., 2006) | |
|  | *bla*_OXA-24_ | OXA-24-F: GGTTAGTTGGCCCCCTTAAA  OXA-24-R: AGTTGAGCGAAAAGGGGATT | | 246 | | 52 | (Woodford et al., 2006) | |
|  | *bla*_OXA-48_ | OXA-48-F: TTGGTGGCATCGATTATCGG  OXA-48-R: GAGCACTTCTTTTGTGATGGC | | 744 | | 52 | (Poirel et al., 2012) | |
|  | *bla*_OXA-58_ | OXA-58-F: AAGTATTGGGGCTTGTGCTG  OXA-58-R: CCCCTCTGCGCTCTACATAC | | 599 | | 56 | (Woodford et al., 2006) | |
|  | *bla*_OXA-143_ | OXA-143-F: TGGCACTTTCAGCAGTTCCT  OXA-143-R: TAATCTTGAGGGGGCCAACC | | 149 | | 52 | (Higgins et al., 2010) | |

**Table S4** **The primers of ESBLs gene segment**

| Target gene | Primer name | Primer sequence（5’—3’） | Product length(bp) | | Annealing temperature  (℃) | | References |
| --- | --- | --- | --- | --- | --- | --- | --- |
| *bla*_CTX-M_ universal | CTX-M-F  CTX-M-R | ATGTGCAGYACCAGTAARGT  TGGGTRAARTARGTSACCAGA | | 593 | | 52 | (Ciesielczuk et al., 2013) |
| *bla*_CTX-M-1_ group | CTX-M-1F  CTX-M-1R | AAAAATCACTGCGCCAGTTC  AGCTTATTCATCGCCACGTT | | 415 | | 52 | (Ciesielczuk et al., 2013) |
| *bla*_CTX-M-2_ group | CTX-M-2F  CTX-M-2R | CGACGCTACCCCTGCTATT  CCAGCGTCAGATTTTTCAGG | | 552 | | 52 | (Ciesielczuk et al., 2013) |
| *bla*_CTX-M-8_ group | CTX-M-8F  CTX-M-8R | TCGCGTTAAGCGGATGATGC  AACCCACGATGTGGGTAGC | | 666 | | 52 | (Ciesielczuk et al., 2013) |
| *bla*_CTX-M-9_ group | CTX-M-9F  CTX-M-9R | ATGGTGACAAAGAGAGTGCA  CCCTTCGGCGATGATTCTC | | 869 | | 52 | (Ciesielczuk et al., 2013) |
| *bla*_CTX-M-25_ group | CTX-M-25F  CTX-M-25R | GCACGATGACATTCGGG  AACCCACGATGTGGGTAGC | | 327 | | 52 | (Ciesielczuk et al., 2013) |
| *bla*_SHV_ | SHV-F  SHV-R | AGCCGCTTGAGCAAATTAAAC  ATCCCGCAGATAAATCACCAC | | 800 | | 52 | (Ciesielczuk et al., 2013) |
| *bla*_TEM_ | TEM-F  TEM-R | CATTTCCGTGTCGCCCTTATTC  CGTTCATCCATAGTTGCCTGAC | | 713 | | 52 | (Ciesielczuk et al., 2013) |
| *bla*_GES_ | GES-ESBL-F  GES-ESBL-R | AGTCGGCTAGACCGGAAAG  TTTGTCCGTGCTCAGGAT | | 399 | | 52 | (Ciesielczuk et al., 2013) |
| *bla*_PER_ | PER-F  PER-R | GCTCCGATAATGAAAGCGT  TTCGGCTTGACTCGGCTGA | | 520 | | 52 | (Ciesielczuk et al., 2013) |
| *bla*_VEB_ | VEB-F  VEB-R | CATTTCCCGATGCAAAGCG  CGAAGTTTCTTTGGACTCTG | | 648 | | 52 | (Ciesielczuk et al., 2013) |
| *bla*_OXA-1_ group | OXA-1-F  OXA-1-R | GGCACCAGATTCAACTTTCAAG  GACCCCAAGTTTCCTGTAAGTG | | 564 | | 52 | (Ciesielczuk et al., 2013) |
| *bla*_OXA-2_ group | OXA-2-F  OXA-2-R | GACCAAGATTTGCGATCAGCAATGCG  CYTTGACCAAGCGCTGATGTTCYACC | | 256 | | 52 | (Ciesielczuk et al., 2013) |
| *bla*_OXA-10_ group | OXA-10-F  OXA-10-R | CGCCAGAGAAGTTGGCGAAGTAAG  GAAACTCCACTTGATTAACTGCGG | | 138 | | 52 | (Ciesielczuk et al., 2013) |

**Table 5 The improved Carba NP system formula**

| System configuration | Reaction mixture（50 μL） | | | | |
| --- | --- | --- | --- | --- | --- |
|  | System 1 | System 2 | System 3 | System 4 | System 5 |
| Imipenem | 0 | 0.15 mg | 0.15 mg | 0.15 mg | 0.15 mg |
| Tazobactam | 0 | 0 | 40 μL | 0 | 40 μL |
| 30 mM EDTA，pH7.8（10×） | 0 | 0 | 0 | 0.1 mL | 5 μL |
| 0.54% phenol red +  1 mM ZnSO4（10×） | 5 μL | 5 μL | 5 μL | 0.1 mL | 5 μL |
| Deionized water | 45 μL | 45 μL | 5 μL | 0.8 mL | 0 μL |
|  |  |  |  |  |  |
| No enzyme production | Red | Red | Red | Red | Red |
| Enzyme A | Red | Yellow | Red | Yellow | Red |
| Enzyme B | Red | Yellow | Yellow | Red | Red |
| Enzyme A and B | Red | Yellow | Yellow | Yellow | Red |
| Unable to determine the type of enzyme production | Other the chromogenic results | | | | |

Table S6 The information of resistance profile and genotype of each isolate

| **Sample** | **Source of sample** | **Cefazolin** | **Ceftazidime** | **Imipenem** | ***bla*_TEM_** | ***Bla***  **_CTX-M_** | ***bla*_SHV_** | ***Bla***  **_OAX-1_** | ***bla*_KPC_** | ***bla*_NDM_** | |
| --- | --- | --- | --- | --- | --- | --- | --- | --- | --- | --- | --- |
| 11001 | faeces | R | R | R | + | + | - | - | - | - | |
| 11002 | faeces | R | R | S | + | + | + | - | - | - | |
| 11003 | faeces | S | S | S | - | - | - | - | - | - | |
| 11004 | faeces | R | S | S | + | + | - | - | - | - | |
| 11005 | faeces | R | S | S | + | - | - | - | - | - | |
| 11006 | faeces | R | R | S | + | + | - | + | - | - | |
| 11007 | faeces | S | S | S | - | - | - | - | - | - | |
| 11008 | faeces | R | R | S | + | + | + | - | - | - | |
| 11009 | faeces | R | R | S | + | - | - | + | - | - | |
| 11010 | faeces | R | R | S | + | + | - | - | - | - | |
| 11011 | urine | R | S | R | + | + | - | - | + | - | |
| 11012 | urine | I | S | S | - | - | - | - | - | - | |
| 11013 | urine | R | S | S | + | + | - | - | - | - | |
| 11014 | urine | R | R | S | + | + | - | + | - | - | |
| 11015 | urine | S | S | S | - | - | - | - | - | - | |
| 11016 | urine | R | R | S | + | + | + | - | - | - | |
| 11017 | urine | R | R | S | + | + | - | + | - | - | |
| 11018 | urine | R | R | S | + | + | + | - | - | - | |
| 11019 | urine | R | S | S | + | + | - | - | - | - | |
| 11020 | urine | S | S | S | - | - | - | - | - | - | |
| 11021 | soil | R | R | S | + | + | + | - | - | - | |
| 11022 | soil | I | S | S | - | - | - | - | - | - | |
| 11023 | soil | S | S | S | - | - | - | - | - | - | |
| 11024 | soil | R | S | S | + | + | - | - | - | - | |
| 11025 | soil | R | S | S | + | + | - | + | - | - | |
| 11026 | soil | I | S | S | - | - | - | - | - | - | |
| 11027 | soil | S | S | S | - | - | - | - | - | - | |
| 11028 | soil | I | S | S | - | - | - | - | - | - | |
| 11029 | water | R | R | S | + | + | + | - | - | - | |
| 11030 | water | S | S | S | - | - | - | - | - | - | |
| 11031 | water | I | S | S | - | - | - | - | - | - | |
| 11032 | water | I | S | S | - | - | - | - | - | - | |
| 11033 | water | S | S | S | - | - | - | - | - | - | |
| 11034 | water | R | S | S | + | + | - | - | - | - | |
| 11035 | water | S | S | S | - | - | - | - | - | - | |
| 15101 | faeces | R | R | S | + | + | - | + | - | - | |
| 15102 | faeces | R | S | S | + | + | - | - | - | - | |
| 15103 | faeces | R | R | S | + | + | - | - | - | - | |
| 15104 | faeces | R | S | R | + | + | - | - | - | - | |
| 15105 | faeces | R | R | S | + | + | - | + | - | - | |
| 15106 | faeces | S | S | S | - | - | - | - | - | - | |
| 15107 | faeces | R | R | S | + | + | - | - | - | - | |
| 15108 | faeces | R | S | S | + | + | - | - | - | - | |
| 15109 | faeces | R | R | S | + | + | + | - | - | - | |
| 15110 | faeces | R | R | S | + | + | - | - | - | - | |
| 15111 | urine | R | S | S | + | + | - | - | - | - | |
| 15112 | urine | R | R | S | + | + | - | - | - | - | |
| 15113 | urine | S | S | S | - | - | - | - | - | - | |
| 15114 | urine | I | S | S | - | - | - | - | - | - | |
| 15115 | urine | R | R | S | + | + | - | - | - | - | |
| 15116 | urine | R | S | S | + | + | - | - | - | - | |
| 15117 | urine | R | R | S | + | + | + | - | - | - | |
| 15118 | urine | R | S | S | - | + | - | - | - | - | |
| 15119 | urine | R | R | S | + | + | - | - | - | - | |
| 15120 | urine | R | S | S | + | + | - | - | - | - | |
| 15121 | soil | I | S | S | - | - | - | - | - | - | |
| 15122 | soil | R | S | S | + | + | + | - | - | - | |
| 15123 | soil | R | R | S | + | + | - | - | - | - | |
| 15124 | soil | I | S | S | - | - | - | - | - | - | |
| 15125 | soil | R | S | S | + | + | - | + | - | - | |
| 15126 | soil | R | S | S | + | + | - | - | - | - | |
| 15127 | soil | S | S | S | - | - | - | - | - | - | |
| 15128 | water | R | R | S | + | + | + | - | - | - | |
| 15129 | water | I | S | S | - | - | - | - | - | - | |
| 15130 | water | S | S | S | - | - | - | - | - | - | |
| 15131 | water | R | S | S | + | + | - | - | - | - | |
| 15132 | water | S | S | S | - | - | - | - | - | - | |
| 15133 | water | I | S | S | - | - | - | - | - | - | |
| 15134 | water | S | S | S | - | - | - | - | - | - | |
| 16211 | faeces | R | R | S | + | + | - | + | - | - | |
| 16212 | faeces | R | S | S | + | + | - | - | - | - |  |
| 16213 | faeces | S | S | S | - | - | - | - | - | - |  |
| 16214 | faeces | R | R | S | + | + | + | - | - | - |  |
| 16215 | faeces | R | S | S | - | + | - | - | - | - | |
| 16216 | faeces | R | R | S | + | + | - | - | - | - | |
| 16217 | faeces | R | S | S | - | + | - | - | - | - | |
| 16218 | faeces | R | R | S | + | + | + | - | - | - | |
| 16219 | faeces | R | S | S | + | + | - | - | - | - | |
| 16220 | faeces | R | R | S | + | + | - | + | - | - | |
| 16221 | urine | R | R | S | + | + | + | - | - | - | |
| 16222 | urine | R | S | S | + | + | - | - | - | - | |
| 16223 | urine | R | R | S | + | + | + | - | - | - | |
| 16224 | urine | S | S | S | - | - | - | - | - | - | |
| 16225 | urine | R | S | S | + | + | - | - | - | - | |
| 16226 | urine | R | R | S | + | + | + | - | - | - | |
| 16227 | urine | I | S | S | - | - | - | - | - | - | |
| 16228 | urine | R | R | S | + | + | - | - | - | - | |
| 16229 | urine | R | R | S | + | + | - | + | - | - | |
| 16230 | urine | R | S | S | + | + | - | - | - | - | |
| 16231 | soil | R | S | S | + | + | - | - | - | - | |
| 16232 | soil | S | S | S | + | - | - | - | - | - | |
| 16233 | soil | R | R | S | + | + | + | - | - | - | |
| 16234 | soil | I | S | S | - | - | - | - | - | - | |
| 16235 | soil | R | S | S | + | + | - | - | - | - | |
| 16236 | soil | R | R | S | + | + | - | + | - | - | |
| 16237 | soil | S | S | S | - | - | - | - | - | - | |
| 16238 | soil | I | S | S | - | - | - | - | - | - | |
| 16239 | water | R | R | S | + | + | + | - | - | - | |
| 16240 | water | R | S | S | + | + | - | - | - | - | |
| 16241 | water | S | S | S | - | - | - | - | - | - | |
| 16242 | water | I | S | S | - | - | - | - | - | - | |
| 16243 | water | I | S | S | - | - | - | - | - | - | |
| 16244 | water | S | S | S | - | - | - | - | - | - | |
| 417741 | faeces | R | R | S | + | + | + | - | - | - | |
| 417742 | faeces | R | S | S | + | + | - | + | - | - | |
| 417743 | faeces | R | R | S | + | + | - | - | - | - | |
| 417744 | faeces | R | S | S | - | + | + | - | - | - | |
| 417745 | faeces | R | S | S | + | - | - | - | - | - | |
| 417746 | faeces | R | R | S | + | + | + | - | - | - | |
| 417747 | faeces | R | S | S | + | + | - | - | - | - | |
| 417748 | faeces | R | R | S | + | + | + | - | - | - | |
| 417749 | faeces | I | S | S | - | - | - | - | - | - | |
| 417750 | faeces | R | R | S | + | + | - | + | - | - | |
| 417751 | urine | R | S | S | + | + | - | - | - | - | |
| 417752 | urine | R | R | S | + | + | + | - | - | - | |
| 417753 | urine | S | S | S | - | - | - | - | - | - | |
| 417754 | urine | R | S | S | + | + | - | - | - | - | |
| 417755 | urine | R | R | S | + | + | + | - | - | - | |
| 417756 | urine | R | R | S | + | + | - | - | - | - | |
| 417757 | urine | R | S | S | + | + | - | + | - | - | |
| 417758 | urine | I | S | S | - | - | - | - | - | - | |
| 417759 | urine | R | R | S | + | + | - | - | - | - | |
| 417760 | urine | R | S | S | + | + | - | - | - | - | |
| 417761 | soil | S | S | S | - | - | - | - | - | - | |
| 417762 | soil | I | S | S | - | - | - | - | - | - | |
| 417763 | soil | R | R | S | + | + | + | - | - | - | |
| 417764 | soil | R | R | S | + | + | - | - | - | - | |
| 417765 | soil | R | S | S | - | + | - | + | - | - | |
| 417766 | soil | I | S | S | - | - | - | - | - | - | |
| 417767 | soil | R | R | S | + | + | - | + | - | - | |
| 417768 | water | S | S | S | - | - | - | - | - | - | |
| 417769 | water | R | S | S | + | + | - | - | - | - | |
| 417770 | water | I | S | S | - | - | - | - | - | - | |
| 417771 | water | S | S | S | - | - | - | - | - | - | |
| 417772 | water | I | S | S | - | - | - | - | - | - | |
| 417773 | water | I | S | S | - | - | - | - | - | - | |
| 417774 | water | S | S | S | - | - | - | - | - | - | |
| 417831 | faeces | R | R | S | + | + | + | - | - | - | |
| 417832 | faeces | R | S | S | + | + | - | - | - | - | |
| 417833 | faeces | R | S | S | + | + | - | + | - | - | |
| 417834 | faeces | R | R | S | + | + | - | - | - | - | |
| 417835 | faeces | R | S | R | + | + | - | + | - | - | |
| 417836 | faeces | S | S | S | - | - | - | - | - | - | |
| 417837 | faeces | R | S | S | + | - | - | - | - | - | |
| 417838 | faeces | R | R | S | + | + | + | - | - | - | |
| 417839 | faeces | R | S | S | + | - | - | - | - | - | |
| 417840 | faeces | R | S | S | + | + | - | - | - | - | |
| 417841 | urine | R | R | S | + | + | - | + | - | - | |
| 417842 | urine | R | S | S | - | - | - | - | - | - | |
| 417843 | urine | R | R | S | + | + | + | - | - | - | |
| 417844 | urine | S | S | S | - | - | - | - | - | - | |
| 417845 | urine | R | S | S | + | - | - | + | - | - | |
| 417846 | urine | R | R | S | + | + | + | - | - | - | |
| 417847 | urine | R | R | S | + | + | - | - | - | - | |
| 417848 | urine | R | R | S | + | + | - | + | - | - | |
| 417849 | urine | R | S | S | + | + | - | - | - | - | |
| 417850 | urine | R | R | S | + | + | - | - | - | - | |
| 417851 | soil | R | S | S | + | + | - | + | - | - | |
| 417852 | soil | R | S | S | + | - | - | - | - | - | |
| 417853 | soil | R | R | S | + | + | + | - | - | - | |
| 417854 | soil | R | R | S | + | + | - | - | - | - | |
| 417855 | soil | I | S | S | - | - | - | - | - | - | |
| 417856 | soil | S | S | S | - | - | - | - | - | - | |
| 417857 | water | R | R | S | + | + | - | + | - | - | |
| 417858 | water | I | S | S | - | - | - | - | - | - | |
| 417859 | water | I | S | S | - | - | - | - | - | - | |
| 417860 | water | I | S | S | - | - | - | - | - | - | |
| 417861 | water | S | S | S | - | - | - | - | - | - | |
| 417862 | water | R | S | S | + | + | - | - | - | - | |
| 417863 | water | S | S | S | - | - | - | - | - | - | |
| 417864 | water | I | S | S | - | - | - | - | - | - | |
| 417950 | faeces | R | R | S | + | + | + | - | - | - | |
| 417951 | faeces | R | R | S | - | + | + | - | - | - | |
| 417952 | faeces | R | S | S | + | + | - | - | - | - | |
| 417953 | faeces | R | S | S | + | - | - | - | - | - | |
| 417954 | faeces | R | R | S | + | + | - | - | - | - | |
| 417955 | faeces | R | S | S | + | - | - | + | - | - | |
| 417956 | faeces | R | S | S | + | + | - | - | - | - | |
| 417957 | faeces | R | R | R | - | + | - | - | - | + | |
| 417958 | faeces | R | R | S | + | + | - | - | - | - | |
| 417959 | faeces | R | S | S | + | + | - | - | - | - | |
| 417960 | urine | R | R | S | + | + | + | - | - | - | |
| 417961 | urine | R | S | S | + | + | - | - | - | - | |
| 417962 | urine | R | R | S | + | + | - | + | - | - | |
| 417963 | urine | R | S | S | + | + | - | - | - | - | |
| 417964 | urine | R | R | S | + | + | + | - | - | - | |
| 417965 | urine | R | S | S | + | + | - | - | - | - | |
| 417966 | urine | R | R | S | + | + | - | - | - | - | |
| 417967 | urine | R | R | S | + | + | - | - | - | - | |
| 417968 | urine | S | S | S | - | - | - | - | - | - | |
| 417969 | urine | R | R | S | + | + | - | - | - | - | |
| 417970 | soil | R | S | S | + | + | - | - | - | - | |
| 417971 | soil | I | S | S | - | - | - | - | - | - | |
| 417972 | soil | I | S | S | - | - | - | - | - | - | |
| 417973 | soil | R | R | S | + | + | + | - | - | - | |
| 417974 | soil | R | R | S | + | + | - | + | - | - | |
| 417975 | soil | I | S | S | - | - | - | - | - | - | |
| 417976 | soil | S | S | S | - | - | - | - | - | - | |
| 417977 | soil | I | S | S | - | - | - | - | - | - | |
| 417978 | water | R | R | S | + | + | - | - | - | - | |
| 417979 | water | R | S | S | + | + | - | + | - | - | |
| 417980 | water | S | S | S | - | - | - | - | - | - | |
| 417981 | water | I | S | S | - | - | - | - | - | - | |
| 417982 | water | S | S | S | - | - | - | - | - | - | |
| 417983 | water | R | S | S | + | + | - | - | - | - | |
| 417984 | water | I | S | S | - | - | - | - | - | - | |

Note: R=Resistant, I=Intermediate, S= Susceptible, +: Positive, -: Negative.

### Figure


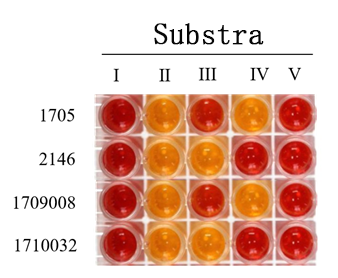


**Figure S1 The result of Carba NP of four isolates.**

**References**

Ciesielczuk, H., Hornsey, M., Choi, V., Woodford, N., and Wareham, D.W. (2013). Development and evaluation of a multiplex PCR for eight plasmid-mediated quinolone-resistance determinants. *J Med Microbiol* 62(Pt 12)**,** 1823-1827. doi: 10.1099/jmm.0.064428-0.

Higgins, P.G., Lehmann, M., and Seifert, H. (2010). Inclusion of OXA-143 primers in a multiplex polymerase chain reaction (PCR) for genes encoding prevalent OXA carbapenemases in *Acinetobacter spp.* *Int J Antimicrob Agents* 35(3)**,** 305. doi: 10.1016/j.ijantimicag.2009.10.014.

Hong, S.S., Kim, K., Huh, J.Y., Jung, B., Kang, M.S., and Hong, S.G. (2012). Multiplex PCR for rapid detection of genes encoding class A carbapenemases. *Ann Lab Med* 32(5)**,** 359-361. doi: 10.3343/alm.2012.32.5.359.

Poirel, L., Potron, A., and Nordmann, P. (2012). OXA-48-like carbapenemases: the phantom menace. *J Antimicrob Chemother* 67(7)**,** 1597-1606. doi: 10.1093/jac/dks121.

Poirel, L., Walsh, T.R., Cuvillier, V., and Nordmann, P. (2011). Multiplex PCR for detection of acquired carbapenemase genes. *Diagn Microbiol Infect Dis* 70(1)**,** 119-123. doi: 10.1016/j.diagmicrobio.2010.12.002.

Voets, G.M., Fluit, A.C., Scharringa, J., Cohen Stuart, J., and Leverstein-van Hall, M.A. (2011). A set of multiplex PCRs for genotypic detection of extended-spectrum beta-lactamases, carbapenemases, plasmid-mediated AmpC beta-lactamases and OXA beta-lactamases. *Int J Antimicrob Agents* 37(4)**,** 356-359. doi: 10.1016/j.ijantimicag.2011.01.005.

Woodford, N., Ellington, M.J., Coelho, J.M., Turton, J.F., Ward, M.E., Brown, S., et al. (2006). Multiplex PCR for genes encoding prevalent OXA carbapenemases in *Acinetobacter spp*. *Int J Antimicrob Agents* 27(4)**,** 351-353. doi: 10.1016/j.ijantimicag.2006.01.004.
